# Comparative study between deep learning and QSAR classifications for TNBC inhibitors and novel GPCR agonist discovery

**DOI:** 10.1038/s41598-020-73681-1

**Published:** 2020-10-08

**Authors:** Lun K. Tsou, Shiu-Hwa Yeh, Shau-Hua Ueng, Chun-Ping Chang, Jen-Shin Song, Mine-Hsine Wu, Hsiao-Fu Chang, Sheng-Ren Chen, Chuan Shih, Chiung-Tong Chen, Yi-Yu Ke

**Affiliations:** Institute of Biotechnology and Pharmaceutical Research, National Health Research Institutes, Zhunan, 35053 Miaoli County Taiwan, ROC

**Keywords:** Biochemistry, Computational biology and bioinformatics

## Abstract

Machine learning is a well-known approach for virtual screening. Recently, deep learning, a machine learning algorithm in artificial neural networks, has been applied to the advancement of precision medicine and drug discovery. In this study, we performed comparative studies between deep neural networks (DNN) and other ligand-based virtual screening (LBVS) methods to demonstrate that DNN and random forest (RF) were superior in hit prediction efficiency. By using DNN, several triple-negative breast cancer (TNBC) inhibitors were identified as potent hits from a screening of an in-house database of 165,000 compounds. In broadening the application of this method, we harnessed the predictive properties of trained model in the discovery of G protein-coupled receptor (GPCR) agonist, by which computational structure-based design of molecules could be greatly hindered by lack of structural information. Notably, a potent (~ 500 nM) mu-opioid receptor (MOR) agonist was identified as a hit from a small-size training set of 63 compounds. Our results show that DNN could be an efficient module in hit prediction and provide experimental evidence that machine learning could identify potent hits in silico from a limited training set.

## Introduction

Implementation of “big data” with deep learning has created a paradigm shift in many scientific disciplines^1–3^. From the perspective of medicinal chemistry, predicting particular functions or properties, e.g., absorption, distribution, metabolism, and excretion (ADME), of a molecular entity might greatly increase the quality of hit compounds and quicken the drug-discovery process. The use of artificial intelligence (AI) in drug design to generate a prediction model, conduct virtual screening, and predict compounds’ activities has received much attention recently^4–7^. Traditionally, quantitative structure–activity relationship (QSAR) model was utilized by medicinal chemists and statisticians to associate bioactivities to particular functional group manipulations. In particular, a linear equation was generated to correlate the features and bioactivities for each compound, while different descriptors were employed to calculate the physical properties to merge with the 3D-structrual information and generate 2D or 3D-QSAR models^8–10^. Nowadays the development of QSAR have apply to multi-target and multi-objective QSAR approaches to assist drug design^11–13^. These QSAR approaches are able to integrate multiple diverse chemical and biological data, being therefore capable of jointly making predictions ranging from in vitro and in vivo activities to ADMET properties^14^. Nonetheless, these QSAR models were hard to generate from random and diverse databases. In addition, to properly separate the training set and the test set was time consuming. To provide an alternative strategy, as reported by Zhavoronkov et al., they have successfully used the deep learning method in the designs of more potent compounds^15^. The incorporation of machine learning method for the progressive analysis of the active compounds and concurrent generation of the prediction model should address such limitations.

Lavecchia et al.^16^ summarized applications of machine learning algorithms, such as support vector machine (SVM)^17^ for ADME evaluation and decision tree (DT) in the classification of compounds^18^. Moreover, a Naïve Bayesian classifier is frequently used in chemoinformatics for predicting biological properties, while *k*-Nearest neighbors (*K*-NN) is a simple and rough method to predict and rank the molecule^19,20^. Others like the artificial neural networks (ANNs), is the popular technique for compound classification, QSAR studies, and primary virtual screening (VS) of compounds^21^. All these machine learning algorithms were programmed to pick out and reclassify important features of the molecules as instructed, the limitations of these algorithms stemmed from the intrinsic inability to “self-taught” and prioritize the features in relation to the activities. Improper combining of the compounds’ descriptors could increase the noise level in features learning that could result in swamping the classifier model and generate a misleading prediction^22^.

Herein, we employed deep learning algorithm to analyze the compound features, generate a first-hand model through 613 descriptors for training, and validated its findings through experimental confirmation. In addition, we compared its accuracy and efficiency with three other different virtual screening methods. After in silico screening of our in-house database of 165,000 compounds, by which different hit compounds were identified from^15,23–25^, 100 top-ranked newly identified TNBC inhibitors were subjected to the bioassay to cross-examine the model accuracy. Moreover, to extend the scope of this deep learning model in predicting meaningful hits, another case study for the search of novel G protein-coupled receptor (GPCR) agonist was carried out. By using a similar model, we only trained the model with a collection of 63 mu-opioid receptor (MOR) agonists to learn the importance of compound features for the given bioactivities. We then identified the nanomolar MOR agonist from the in-house compounds library. Our study suggested that deep learning method could generate potent hit compounds in different disease areas for the drug discovery process.

## Results and discussion

### Model generation and comparative studies in efficiency

An advancement in the virtual screening method was made to reduce the burden of the drug discovery/development processes in a cost-effective manner^26^. The virtual screening can be devised by using either structure-based virtual screening (SBVS) like docking screening methods^27^ or LBVS like QSAR model screening^28^. To investigate the application and efficiency of the DNN approach in medicinal chemistry, we compared other contemporary QSAR method, such as RF approach^29^, with traditional QSAR methods, such as PLS and MLR. RF has been demonstrated to have high prediction accuracy and robustness with adjustable parameters. It has become a “gold standard” machine learning method. Meanwhile, partial least squares (PLS) and multiple linear regression (MLR) are methods used for large data manipulation and allow facile generation of the model unlike other 3D-QSAR methods. In the current study, the same data set and descriptors were systematically incorporated to generate the models.

The traditional QSAR model helps to identify the relationship between activities and the descriptors’ variables. In addition to the QSAR methods, RF and DNN from the machine learning approach were used to generate the prediction model. RF is an ensemble learning method to perform classification in a similar manner to that of the decision tree (DT). Yet, the major difference stems from the use of Bagging method (or Bootstrap Aggregating) to generate many individual trees^30^. Each tree could self-process samples from the training set data and provide a fixed number of random sampling data from the training set to generate a DT for voting. The final model was based on the highest score from individually developed trees in the forest. On the other hand, DNN are mathematical methods developed to mimic the neurons (nodes) of the human brain to recognize objects and analyze progressively, improving the efficiency of previously reported neural network algorithms^1,31^. Each neuron is treated as a particular feature to classify the complex factors. The system, in turn, learns from the training set and assigns different weights for each neuron as this model eventually facilitates a prediction following the different clusters. Taken together, DNN increase the hidden layer numbers by allowing each layer of the nodes to access different features based on the previous layer’s output. Consequently, as more executed nodes are added in each layer, more features are recognized, enhancing the overall decision process.

**Figure 1:**
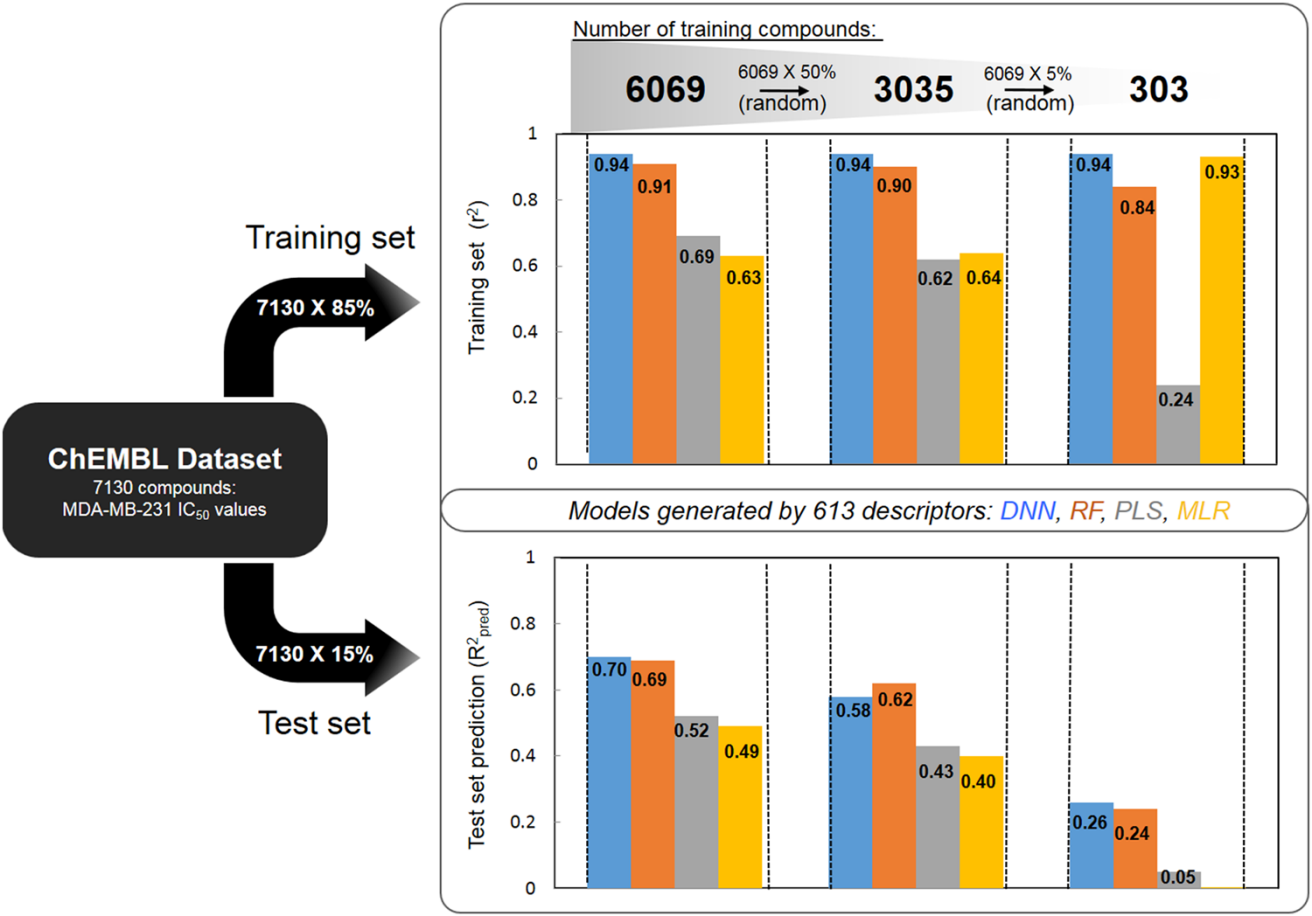
Comparative studies of classification methods. Models generated through 613 descriptors were trained and tested using the ChEMBL dataset of 7130 compounds that exhibited MDA-MB-231 IC_50_ values. The training and test sets’ prediction efficiencies between different models, DNN, RF, PLS, and MLR were compared with decreasing number of training compounds.

To compare the different methods of virtual screening, a database of 7130 molecules with previously reported MDA-MB-231 inhibitory activities were collected from the ChEMBL web service. As the model prediction accuracy is highly depended on the quality of the database. In this study, these compounds were then randomly separated into 6069 compounds (the training set) and 1061 compounds (the test set) to evaluate which model can more efficiently analyze the database and generate more useful models (Fig. 1). We implemented the extended connectivity fingerprints (ECFPs), which are circular topological depictions of the molecules, as the major molecular descriptors. Specifically, ECFPs are generated in a molecule-directed manner by systematically recording the neighborhood of each non-hydrogen atom into multiple circular layers up to a given diameter of that molecule^32^. These atom-centered sub-structural features are then mapped into integer codes and the resulting identifiers shape the extended connectivity fingerprint. These identifiers capture the local information of the corresponding atom in such a way that various atom properties (e.g., atomic number, connection counts) are packed into a single integer value. The default identifier configuration of ECFP captures highly specific atomic information, enabling the representation of a large set of precisely defined structural features.

In some applications, however, different kinds of abstraction may be desirable. For example, a chlorine or a bromine substituent on a ring may be functionally equivalent but would be redundantly distinguished by ECFP. Alternatively, functional-class fingerprints (FCFPs)^32^ detail circular fingerprints via the pharmacophore identification of atoms, which reports topological pharmacophore fingerprints. To perform the classifications comparisons, the software devised a total of 613 descriptors from AlogP_count^33^, ECFP, and FCFP to generate the model (Fig. 1, and supplementary data Table S1).

Three distinct different numbers of training set (6069, 3035, and 303 compounds) were used to generate the models and their efficiencies were evaluated by the fixed test set (1061 compounds). R-square value (r^2^ value) was used to quantify the differential efficiencies between the training set and test set prediction in machine learning methods (DNN and RF) and the QSAR methods (PLS and MLR) (Fig. 1). With training set compounds fixed at 6069, the machine learning methods (DNN or RF) exhibited higher predicted r^2^ value near 90% than the traditional QSAR method (PLS or MLR) at 65%. In general, a good model was considered as having larger r^2^ and R^2^_pred_ (r^2^ > 80, R^2^_pred_ > 60 is an assessable model)^34–36^. With the decrease of training set numbers, the machine learning methods sustained the overall higher r^2^ value. As the training set number decreases, the deviation only retained with DNN and RF at 0.84 to 0.94, while PLS and MLR dropped to 0.24 from 0.69. In particular, with significantly lower training set numbers, interestingly, the MLR method maintained a respectful r^2^ value near 0.93, but when running against the test set, R^2^_pred_ ${\mathrm{R}}_{\mathrm{pred}}^{2}$ was calculated to be zero. This implies that MLR could be an over-fitting model with a high false-positive rate, especially when the numbers of learning compounds are very limited. These results showed that the PLS and MLS methods could not efficiently distinguish the descriptors and were problematic in generating meaningful fitting equations. On the other hand, the DNN method with lower number of training sets, the data still held a higher r^2^ value of 0.94 than that of 0.84 by RF method (Fig. 1). Although the RF method could classify the features and select intrinsic feature for the analysis, DNN method was better in providing insights in weighting of important features. As a result, the DNN method held a higher r^2^ value with lower numbers of training data sets. Of the machine learning methods, the R^2^_pred_ ${\mathrm{R}}_{\mathrm{pred}}^{2}$ significantly improved with the increase in training set numbers, which is vastly different than the QSAR models (Fig. 1). With routine sampling of large amount of molecular features against a target from the public domain might be limiting, the large spread or deviation of PLS and MLR processes could greatly hinder the potential of identifying potent hits. Taken together, DNN and RF exhibited better accuracy and efficiency in the prediction of hit compounds. As shown in Fig. 1, the R^2^_pred_ of DNN (0.26) and RF (0.24) were much lower, which implies that the database quality might not be sufficient for learning. We envision that more datasets might be needed or the quality of the datasets in terms of structural information and their activities should be more correlated for better learning by the algorithm.

Seminal work by Grisoni and coworkers^37,38^, have indicated the R^2^_pred_ or Q^2^ metrics (Eq. 1) should be optimize to ${\mathrm{Q}}_{F3}^{2}$ (Eq. 2) as it was more sensitive for comparing predicted abilities between different models with the same training set. The original R^2^_pred_ metrics was shown bellow

1 $${\mathrm{Q}}^{2}=1-\frac{\sum_{i=1}^{{n}_{test}}{({y}_{i}-{\widehat{y}}_{i})}^{2}}{{\sum_{i=1}^{{n}_{test}}({y}_{i}-{\stackrel{-}{y}}_{TR})}^{2}}$$

where y_i_ is the experimental result for i-th compounds not existing in the training set, ŷ_i_ is the predicted result of the i-th compound, y̅_TR_ is the average value of the training set experimental results, and n_test_ is the test set numbers. Reported by Todeschini et al., the ${\mathrm{Q}}_{F3}^{2}$ should be calculated as

2 $${\mathrm{Q}}_{F3}^{2}=1-\frac{\sum_{i=1}^{{n}_{test}}{\left({y}_{i}-{\widehat{y}}_{i}\right)}^{2}/{n}_{test}}{{\sum_{j=1}^{{n}_{TR}}({y}_{j}-{\stackrel{-}{y}}_{TR})}^{2}/{n}_{TR}}$$

By which, y_j_ is the experimental result for training set , y̅_TR_ is the average value of the training set experimental result, and n_TR_ is the training set numbers. By applying this metric to our studies, the DNN and RF exhibited highest ${\mathrm{Q}}_{F3}^{2}$ value of 0.679 and 0.670, respectively (Supplementary data Table S2). In addition, Consonni et al. showed the calculation of Root-Mean-Square Error in prediction (RMSEP) and Root-Mean-Square Error in calculation (RMSEC) could quantify predictive abilities of QSAR model. The higher value of RMSEP led to higher chances of error. Our calculation results also showed that DNN method had the lowest value for RMSEC and RMSEP in comparison to those of other models (Supplementary data Table S2).

**Figure 2:**
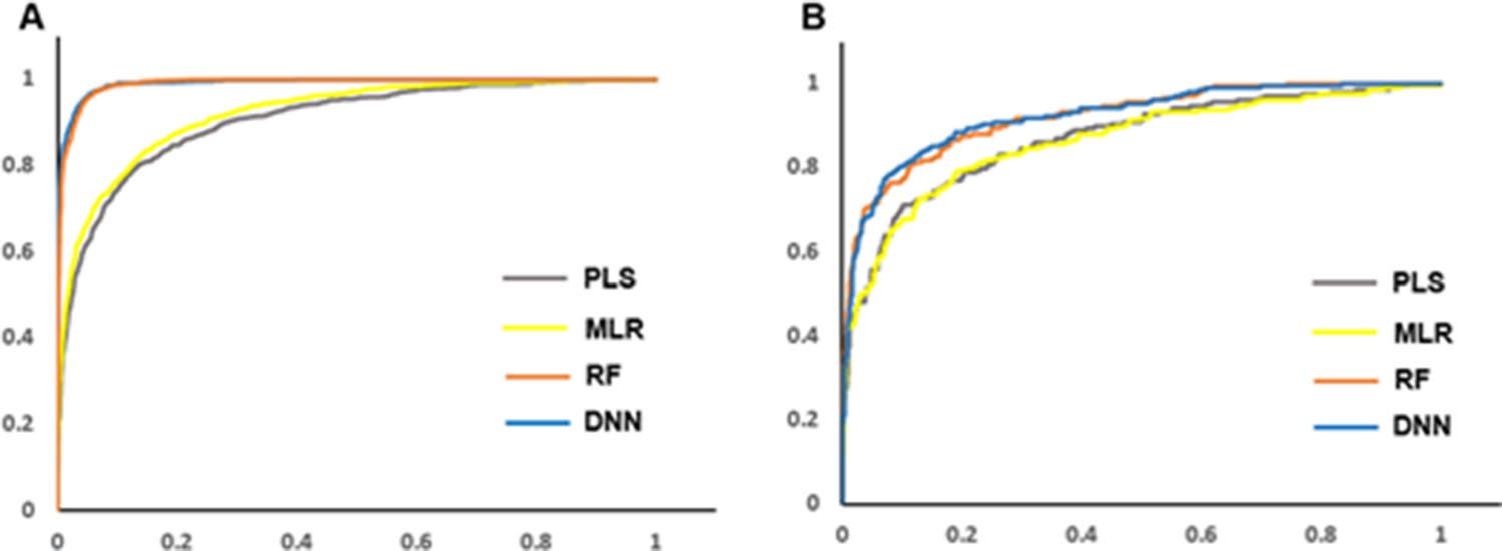
Comparative studies of ROC calculation for DNN, RF, PLS, and MLR methods. Comparisons of ROC curves for (**A**) Using the fix number of training set (6069 compounds) to generate the model for the analysis of the training set itself. The AUC value of each ROC curve for PLS, MLR, RD, and DNN are 0.907, 0.922, 0.991, 0.922, respectively. (**B**) Using the fix number of training set (6069 compounds) to generate model for analysis of the test set. The AUC value of each ROC curve for PLS, MLR, RD, and DNN are 0.924, 0.922, 0.870, 0.865, respectively.

To further investigate the advantageous prediction ability of machine learning methods (DNN and RF) over the traditional QSAR methods (PLS and MLR), we analyzed the receiver operating characteristic (ROC) curve with the fix training set (6069 compounds) and fix test set (1061 compounds)^39,40^. ROC curve evaluates the performance of a binary classifier system and provides means in selecting optimal models. ROC curve was constructed by plotting a graph of sensitivity (Se, true positive rate) vs. 1-specificity (1-Sp, false positive rate). The measure of Se and Sp are defined as

3 $$\mathrm{Se}=\frac{\mathrm{TP}}{\mathrm{TP}+\mathrm{FN}}$$

4 $$\mathrm{Sp}=\frac{\mathrm{TN}}{\mathrm{TN}+\mathrm{FP}}$$

where TP is the number of correctly identified active ligands (true positives), TN is the number of correctly identified inactive ligands (true negatives), FP the number of incorrectly identified active ligands (false positives), and FN the number of incorrectly identified inactive ligands (false negatives). The area under the ROC curve (AUC) measures the performance of each virtual screening approaches. The ideal screening method results in an AUC value of 1, while a random screening method would lead to an AUC value of 0.5. As shown in Fig. 2A, the AUC calculated by the training set of the RF and DNN methods were 0.991 and 0.992, respectively. Interestingly, these values were higher than those of PLS and MLR methods with 0.907 and 0.922. To investigate the prediction ability of the test set, the respective AUC values of RF and DNN methods were 0.922 and 0.924. Also, they were expected to be superior than those of PLS and MLR methods with 0.870 and 0.865. These ROC curve analyses further potentiated the RF and DNN screening method might be more suitable than traditional QSAR methods (PLS and MLR).

### Virtual screening and identification of TNBC inhibitors by DNN and RF models with experimental validation

**Figure 3:**
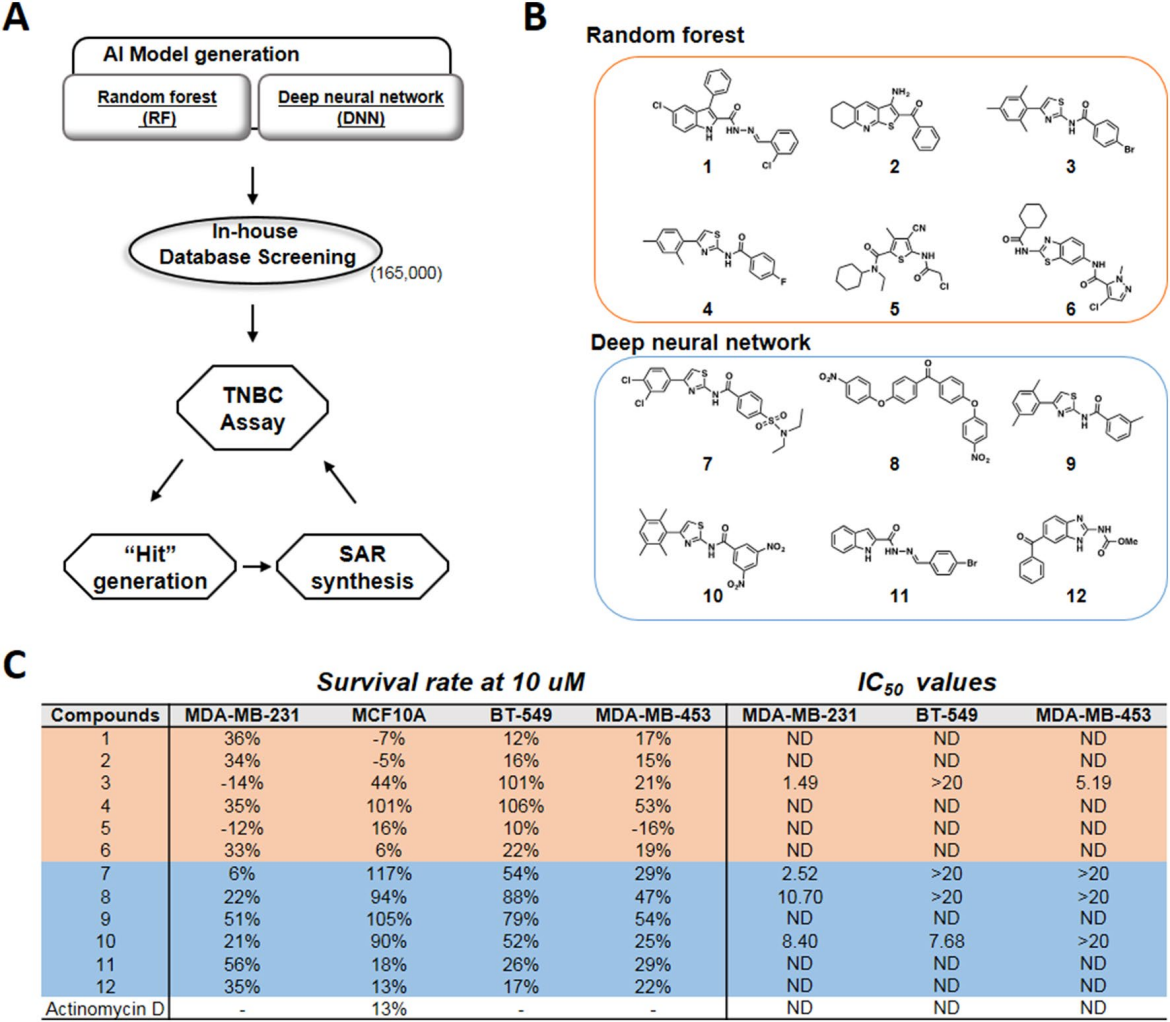
Model generation and discovery of TNBC inhibitors from in-house library. (**A**) Flow scheme of discovery of potent TNBC inhibitors. (**B**) Chemical structures of in-house identified TNBC inhibitors: 6 hits from random forest classifications and 6 hits from deep neural network. (**C**) Cellular survival rate at 10 µM of the 12 hits against nonmalignant mammary epithelial cell line (MCF10A) and three TNBC cell lines: MDA-MB-231, BT-549, and MDA-MB-453. Actinomycin D, was used as control. Values are expressed as the mean of at least two independent determinations and are within ± 15%.

**Figure 4:**
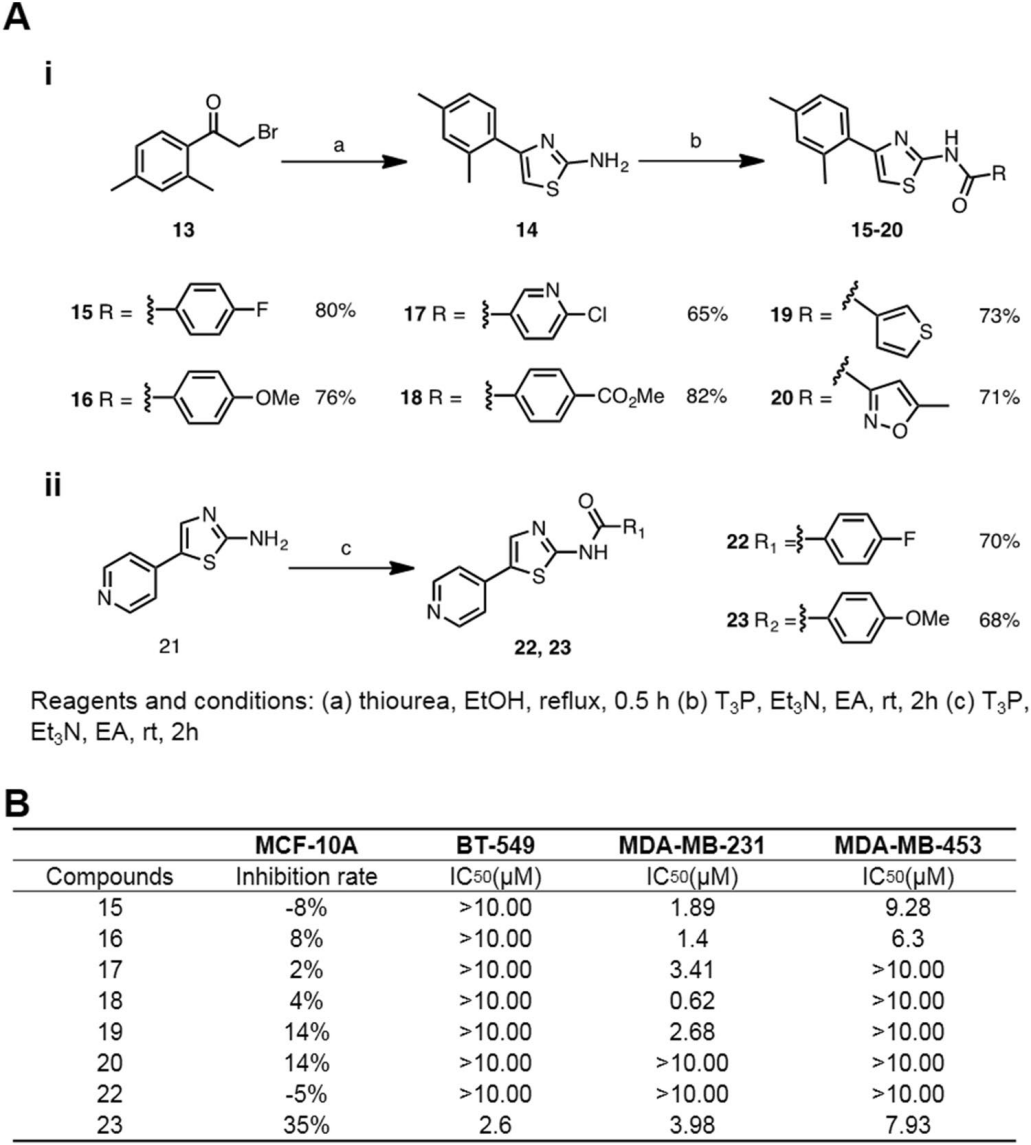
Structure–activity relationship studies of thiazole-based TNBC inhibitors. (**A**) Synthetic routes of thiazole (i) and its regioisomers (ii). (**B**) Cellular cytotoxicity of the inhibitors against nonmalignant mammary epithelial cell line (MCF10A) at 10 µM and IC_50_ values against three TNBC cell lines: MDA-MB-231, BT-549, and MDA-MB-453.

Based on the above information, the DNN and RF models were chosen as the preferred means to perform virtual screening. The identified compounds were then assayed for their corresponding bioactivities. Herein, we demonstrated two different cases for evaluating these models’ accuracy. First, we successfully identified active hits for TNBC inhibition. The DNN and RF models were used to screen the in-house database (165,000 compounds), and the selected hits were assayed against the anti-TNBC cellular assay (Fig. 3A). The top predicted 100 compounds were selected and tested at 10 μM concentration for MDA-MB-231 cell line inhibition (Supplementary data Table S3.1 and Table S3.2). Since the compound collection was acquired based on MDA-MB-231 inhibitory activities, other TNBC cell lines were also assayed to obtain selective TNBC inhibitors. Out of the multiple hits identified through both methods, six compounds from each classification (compounds **1–12**) were assayed and showed low cytotoxicity to MCF10A, a nonmalignant mammary epithelial cell line (Fig. 3B,C). We then assayed these hits against other TNBC cell lines, BT-549 and MDA-MB-453. Compounds **3, 7**, **8**, **10,** which exhibited broader TNBC inhibitions, were then subjected to IC_50_ determination (Supplementary data Figure S1). Notably, between RF and DNN, we obtained a thiazole core with selective anti-TNBC profiles over the normal mammary cells (Fig. 3B,C). Synthesis of the thiazole-based inhibitors were carried out and several potent TNBC inhibitors were identified (Fig. 4). Compound **18**, which showed good selectivity over nonmalignant mammary epithelial cell, had an IC_50_ of 0.62 µM against MDA-MB-231. Interestingly, regioisomeric controls in compounds **22** and **23** were synthesized. Compound **22** did not show activities toward the TNBC and **23**, although it possessed moderate micro molar activities and also exhibited cytotoxicity to MCF-10A. This study serves as a good example of hit generation from an unknown target with good cellular selectivity and functional manipulatable core.

### Analysis between model-identified compounds and database compounds

**Figure 5:**
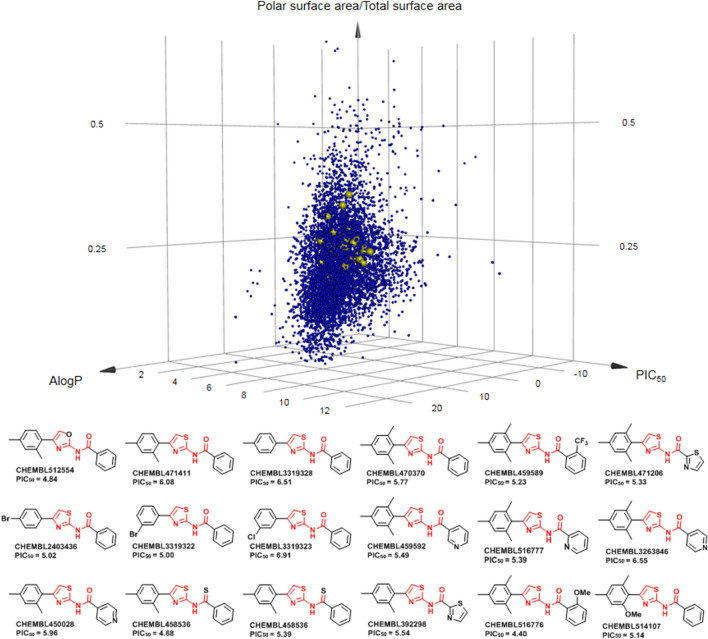
Chemoinformatics of thiazole-based inhibitors in the ChEMBL dataset. Analysis of compound properties is characterized by AlogP, PIC_50_, and percent polar surface area of the molecules to address solubility, potency, and cellular properties. The thiazole compounds were clustered in the center of the matrix.

To address the availability of thiazole core in the original set of 7130 compounds, we devised a principle component analysis of the database with PIC50, AlogP, and polar surface versus total surface area (Fig. 5). These properties were chosen to fulfill the characteristics of a hit compound in a common drug-discovery campaign. The 7130 compounds were then mapped and showed that compounds consisting of the thiazole core are clustered in the quadrant with activities ranging from PIC50 4.8–6.5 (10 µM to 0.3 µM). Moreover, the AlogP and polar surface versus total surface area values were in the satisfactory range for a hit compound (Fig. 5). Gratifyingly, this finding correlates well to the experimental results from our SAR studies of the TNBC inhibitors (Fig. 4). Our findings suggest that both RF and DNN can be adapted to generate meaningful models and identify functional hits for the later optimization process.

### Identification and experimental validation of novel GPCR agonists by the DNN and RF models

**Figure 6:**
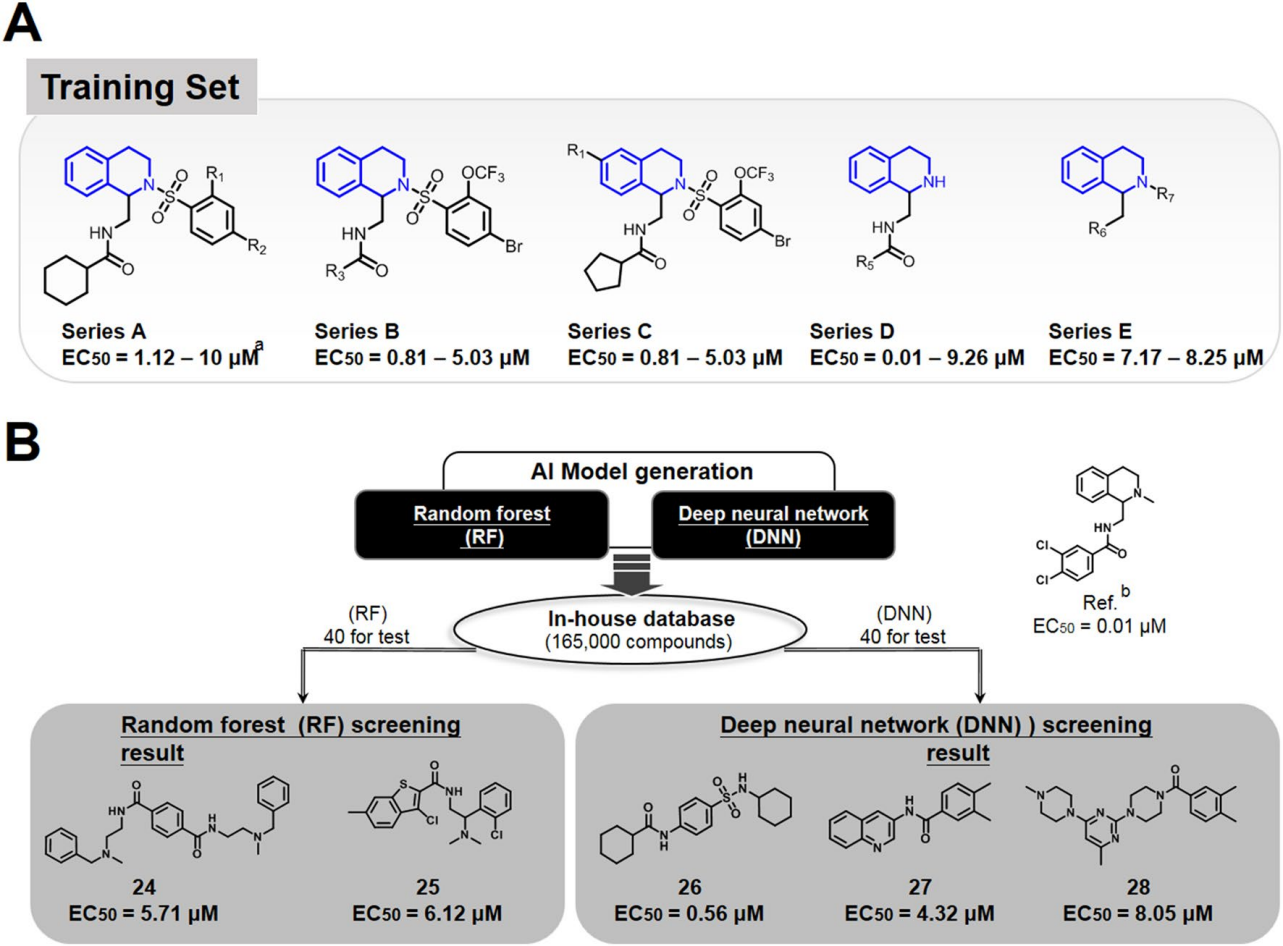
Prediction and validation of novel MOR agonist from RF and DNN classifications. (**A**) Molecular descriptions of training set compounds of MOR agonist, Series A–E, and their corresponding EC_50_ values. (**B**) Flow scheme of model generation and novel hits identified from RF and DNN prediction. By FLIPR calcium assay, EC_50_ values are the means of at least three independent experiments. Reference compound was published by Chen et al. and assigned as compound **46** in the publication.

We envision that predicting new scaffold with the experimental validation should render the greatly expand the application of this deep learning approach. We then adapted this classification for GPCR agonist generation, where structure-based designs are limited without a known information of the core structure due to the membrane associated nature of many GPCRs (Fig. 6). To evaluate the scope of the model, the MOR agonist was also identified via virtual screening of the same in-house database with the DNN and RF models. In our previous studies on MOR agonist^41^, we synthesized 63 compounds and tested by FLIPR calcium assay (Supplementary data Table S4 and Figure S2). We used MOR as an example to demonstrate the predictive power of this approach. To train the learning system, we provided a small sample collection of 63 compounds^41^. The total 63 compounds, divided into series A–E clusters, were used as the training set to generate the DNN and RF models (Fig. 6A). We envision that incorporation of molecular diversity with large spread of bioactivities in series A–E should minimize deviation of the r^2^ with DNN and RF and improve the learning process. Model generation was performed with the same 613 descriptors, and then new cores in the 165,000 in-house pool were processed. The top 40 compounds predicted by RF and another top 40 by DNN (Supplementary data Table S5.1 and Table S5.2) were subjected to the FLIPR calcium assay (Fig. 6B). The CHO-K1 cell line, stably expressing MOR and Gα15 (GenScript), was used to evaluate the selected compounds. In the FLIPR calcium assay of CHO-K1/MOR/Gα15 cells, activation of MOR elicits an intracellular calcium release, leading to an increase in the relative fluorescence units (RFU). Five compounds, **24–28**, were identified as potential hits by these two different screening models. As shown in Fig. 6B, in addition to hit **26** identified from DNN method exhibited potent agonist activities (EC_50_ = 560 nM), these models provided great molecular diversities over the training set of compounds. To the best of our knowledge, this is the first example correlating prediction and validation of a GPCR agonist discovery where structure-based design is limited. Notably, only a small training set of 63 compounds (Supplementary data Table S4) was employed, and a set of five structurally distinct hits was identified. This result provided strong support in that DNN and RF methods could still sustained high predicted r^2^ value in low numbers of training data set.

The Opioid receptor binding affinity assay was performed to further confirm these compounds direct bind to MOR. The MOR membranes was detected by measuring the competitive inhibition ratio of [3H]diprenorphine binding assessment. Ki = IC50/(1 + L/Kd), where L is the concentration of [3H]diprenorphine (1 nM) used, and the Kd value in MOR is 0.4 nM. All assays were carried out independently and at least in triplicate. The values indicate the mean ± SEM. MOR = mu opioid receptor; ME = [Met5]Enkephalin; N.D. = not determined; SEM = standard error of the mean. As shown in Fig. 6, the compounds **24**–**28** has no structural similarity to morphine or any other previously described opioid receptor agonist. In the receptor binding assay, membrane proteins from HEK-MOP were used for detecting the binding affinity of these compounds by comparing with the morphine (Table 1).

**Table 1:** The binding affinity assay of compounds **24, 25**, **26**, **27**, **28** and morphine on MOR.

	[^3^H]diprenorphine binding, Ki (nM)
Morphine	6 ± 1.0
ME	3.5 ± 0.8
24	830 ± 50.0
25	625 ± 79.0
26	535 ± 50.0
27	720 ± 95.0
28	965 ± 110.0

[Met5] Enkephalin (ME) is an opioid pentapeptide.

## Conclusion

Hit identification is an important step in the early stages of drug discovery. Virtual screening is extensively used to identify suitable hits, and such methods to improve the hit rate are much sought after. In this study, we report comparative studies between traditional QSAR methods and machine learning methods applied in VS. The results showed that machine learning methods could achieve a higher predicted r^2^ value with fewer compounds required in the training set. In our work, DNN and RF predicted the selective TNBC inhibitors from the our in-house database. In case of identifying novel MOR agonist, 5 hit compounds were readily found from only a 63-compound training set. The diversified chemical structures of the 5 hits identified by the DNN method showed good potency as a hit with an EC_50_ = 560 nM. This is an interesting application of the deep learning classification as structure-based design of GPCR agonist are limited with limited information of the core structure due to the membrane associated nature of many GPCR. Taken together, this study demonstrated the efficiency of DNN and RF machine learning methods for VS and provided experimental evidences that this application can be adapted to identify hit compounds among different diseases.

## Experimental procedures

### Data set collection for TNBC and MOR

For the TNBC inhibitor identification studies, 7130 compounds that contain MDA-MB-231 bioassay activity data were collected from the ChEMBL database (https://www.ebi.ac.uk/chembl/). The database was randomly separated into two parts. One part contained 85% of the compounds (6069 compounds), which were used as the training set; the other 15% of compounds (1061 compounds) were used as a test set in our studies. However, for the MOR agonist discovery studies, 63 compounds were collected from the publication of Chen et al.^41^ as a training set database (Supplementary data Table S4).

### Descriptors and model generation

All models were generated by BIOVIA pipeline pilot V18.1 platform with R statistic software V 3.4.1^42,43^. These models were generated by the same descriptors from the Discovery Studio/Calculates ligand properties program (BIOVIA, Inc., San Diego, CA), including ALogP_count (101 descriptors), ECFP_4 (256 descriptors), and FCFP_4 (256 descriptors). The RF model use a recursive partitioning (decision tree) forest model by R package “"randomForest". The number of trees was set for 500. The fraction of descriptors to use for each tree in the forest was set to 0.3. A deep neural network model using R package “deepnet” performed the DNN model. Three hidden layers were used and each layer with 80 notes. The learning rate of every epoch was 0.1 with the momentum for 0.9, the maximum number of iterations for network training was 5000. To prevent the model + over-fitting, the fraction of hidden layer to be dropped out for model training was set for 0.25. The traditional QSAR model, like multiple linear regression analysis (MLR), is a equation to describe the dependent variable Y with independent variables, X1, X2, …, etc. For example, Y(pred)^i^ = b0 + b1 * X1 + b2 * X2 + ....+  bp*Xp, where the b1, b2,…,bn are the regression coefficients, Y(pred)^i^ can be project as ith bioactivities, and X1, X2,…,Xp can apply to different descriptors^44^. The PLS regression is using the orthogonal matrices (T) to determine the fundamental relations between dependent variable Y and independent variables X. For example, Y = X × W × Q + E, T = X × W, where Y is a response matrix for the dependent variables like bioactivities result, T is a extraction matrix for the independent variables like descriptors, Q is a matrix of the regression coefficients, W are the factor score matrix and the weight matrix, and E is an error term for the model^45,46^.The PLS and MLR models were also conduct by pilot V18.1 platform with the default protocol and evaluate by fivefold cross-validated method.

### Cell viability assay for TNBC inhibitors

The cells were seeded in 384-well clear plates with a density of 8 × 10^2^ cells/well for MCF-10A and BT-549 cell lines, 1 × 10^3^ cells/well for MDA-MB-453, and 2 × 10^3^ cells/well for MDA-MB-231 overnight. Then cells were treated with the indicated concentrations of test compounds for 72 h. At the end of incubation, 5 μL of PrestoBlue Cell Viability Reagent (Invitrogen, Carlsbad, CA, USA) was added to each well with 50 μL medium. The plates were incubated for an additional 1.5 h at 37 °C in a humidified 5% CO_2_ atmosphere; the relative fluorescence unit (RFU) in the reaction mixture will then be recorded (Ex560/Em590) by **Victor**^**2**^**-V**plate reader (PerkinElmer, Waltham, MA, USA). The cell lines were chosen based on the mutation status of PTEN and/or TP53: MCF-10A, the nonmalignant mammary epithelial cell line; BT-549 with mutation of PTEN and TP53; MDA-MB-453 with mutation of PTEN; MDA-MB-231 with mutation of TP53^47^.

### FLIPR calcium assay

Black with clear flat bottom 96-well assay plates (Corning) were coated with a 0.1 mg/mL Poly-l-Lysine solution a day prior to the assay. CHO-K1/MOR/Gα15 cells were suspended in the F12 medium and plated at a density of ~ 8 × 10^4^ cells/well in 200 μL medium. Cells were incubated in a humidified atmosphere of 10% CO_2_ at 37 °C overnight to reach an 80–90% confluence cell monolayer before assay. On the day of assay, 150 μL medium/well was removed from the plate. To each well, 50 μL FLIPR calcium assay reagent dissolved in 1 × assay buffer (HBSS: KCl 5 mM, KH_2_PO_4_ 0.3 mM, NaCl 138 mM, NaHCO_3_ 4 mM, Na_2_HPO_4_ 0.3 mM, d-glucose 5.6 mM, with an additional 20 mM HEPES and 13 mM CaCl_2_, pH 7.4), with 2.5 mM probenecid added; then the plate was incubated at 37 °C for 1 h. Compounds (30 μM) and other reagents were dissolved in the assay buffer. Using a FlexStationIII (Molecular Devices Corp.), the increase of fluorescence after robotic injections of compounds or other reagents were monitored every 1.52 s interval with excitation wavelength at 485 nm and emission wavelength at 525 nm. The [Ca^2+^]_*i*_ fluorescence was measured up to 90 s after agonist injection. The relative fluorescence intensity from 2 wells of cells were averaged and the relative amount of [Ca^2+^]_*i*_ release was determined by integrating the area under the curve (AUC) with Prism software (GraphPad). The AUC of each compound was then subtracted from the response in the presence of MOR agonist naloxone (20 nM) to obtain the specific MOR responses^48^.

### Radioligand binding assay

Human embryonic kidney 293 cells constitutively expressing MOR (HEK-MOR) (Dr. Ping-Yee Law; University of Minnesota Medical School) were harvested and homogenized in membrane preparation buffer (50 mM Tris–HCl at pH 7.4, containing 2 mM ethylenediaminetetraacetic acid [EDTA]) containing a fresh protease inhibitor cocktail (Roche, Basel, Switzerland) and then centrifuged at 30,000*g* for 30 min. The pellets were resuspended, aliquoted, and stored at − 80 °C. For the [^3^H]diprenorphine saturation binding assays, membranes (containing 25 μg of protein) were incubated with different concentrations (0.5–5 nM) of [^3^H]diprenorphine in binding buffer (50 mM Tris–HCl at pH 7.4, containing 2 mM EDTA) at 25 °C for 1 h. For the competitive binding experiments, [^3^H]diprenorphine (1 nM) was incubated with membranes (containing 25 μg of protein) in the absence or presence of various concentrations of compounds at 25 °C for 1 h. The samples were then rapidly filtered onto glass-fiber filters (Millipore, Billerica, MA, USA) and washed three times with ice-cold phosphate-buffered saline. The radioactivity was quantified using a liquid scintillation counter^49^.

## Supplementary information

Supplementary Information 1.

## Abbreviations

DNN: Deep neural networks
LBVS: Ligand-based virtual screening
RF: Random forest
TNBC: Triple-negative breast cancer
GPCR: G-protein-couple receptors
AI: Artificial intelligence
QSAR: Quantitative structure–activity relationship
SVMs: Support vector machine
ADME: Absorption, distribution, metabolism, and excretion
DT: Decision tree
K-NN: K-nearest neighbors
ANNs: Artificial neural networks
VS: Virtual screening
MOR: Mu-opioid receptor
SBVS: Structure-based virtual screening
PLS: Partial least squares
MLR: Multiple linear regression
ECFPs: Extended connectivity fingerprints
FCFPs: Functional-class fingerprints
FRU: Relative fluorescence units
