# Supplementary material for: Comparative study between deep learning and QSAR classifications for TNBC inhibitors and novel GPCR agonist discovery
Source: Sci Rep. 2020 Oct 8;10:16771. doi: 10.1038/s41598-020-73681-1 (PMC7545175; doi:10.1038/s41598-020-73681-1)

**Supporting Information**

**Comparative study between deep learning and QSAR classifications for TNBC inhibitors and novel GPCR agonist discovery**

Lun K. Tsou^†^, Shiu-Hwa Yeh^†^, Shau-hua Ueng^†^, Jen-Shin Song, Chun-Ping Chang, Mine-Hsine Wu, Hsiao-Fu Chang, Sheng-Ren Chen, Chuan Shih, Chiung-Tong Chen, Yi-Yu Ke*

Institute of Biotechnology and Pharmaceutical Research, National Health Research Institutes, Zhunan, Miaoli County 35053, Taiwan, R.O.C.

**Table of Contents**

**Table S1.** The 613 learning descriptors --------------------------------------------------------S3

**Table S2.** The calculation of $Q_{F3}^{2}$, RMSEC, RMSEP for RF, DNN, PLS, and MLR models with different training set.--------------------------------------------------------------S10

**Table S3.1** The screening TNBC inhibitors identify by RF models. ----------------------S11

**Table S3.2** The screening TNBC inhibitors identify by DNN models. --------------------S16

**Table S4.** The 63 compounds of the MOR agonist. -------------------------------------------S22

**Table S5.1** The screening MOR agonist identify by RF models. --------------------------S27

**Table S5.2** The screening MOR agonist identify by DNN models. -----------------------S29

**Figure S1.** The IC50 calculation from the does-dependence test for TNBC cell-based assay.------------------------------------------------------------------------------------------------S31

**Figure S2**. The EC50 calculation from the does-dependence test for FLIPR calcium assay.--------------------------------------------------------------------------------------------------------S32

The synthesis and identification of the 63 training set compounds. ------------------------S33

The synthesis of TNBC inhibitors. --------------------------------------------------------------S39

The relative NMR , Ms or purity data for the active compounds------------------------------S45

**Table S1. The 613 learning descriptors**

**Descriptors**:

**ALogP_Count**

ALogP_Count.1.,ALogP_Count.2.,ALogP_Count.3., ALogP_Count.4., ALogP_Count.5.,

ALogP_Count.6., ALogP_Count.7., ALogP_Count.8., ALogP_Count.9.,

ALogP_Count.10., ALogP_Count.11., ALogP_Count.12., ALogP_Count.13.,

ALogP_Count.14., ALogP_Count.15., ALogP_Count.16., ALogP_Count.17.,

ALogP_Count.18., ALogP_Count.19., ALogP_Count.20., ALogP_Count.21.,

ALogP_Count.22., ALogP_Count.24., ALogP_Count.25., ALogP_Count.26.,

ALogP_Count.27., ALogP_Count.28., ALogP_Count.29., ALogP_Count.30.,

ALogP_Count.31., ALogP_Count.32., ALogP_Count.33., ALogP_Count.34.,

ALogP_Count.35., ALogP_Count.36., ALogP_Count.37., ALogP_Count.38.,

ALogP_Count.39., ALogP_Count.40., ALogP_Count.41., ALogP_Count.42.,

ALogP_Count.43., ALogP_Count.44., ALogP_Count.46., ALogP_Count.47.,

ALogP_Count.48., ALogP_Count.49., ALogP_Count.50., ALogP_Count.51.,

ALogP_Count.52., ALogP_Count.53., ALogP_Count.54., ALogP_Count.56.,

ALogP_Count.57., ALogP_Count.58., ALogP_Count.59., ALogP_Count.60.,

ALogP_Count.61., ALogP_Count.62., ALogP_Count.64., ALogP_Count.66.,

ALogP_Count.67., ALogP_Count.68., ALogP_Count.69., ALogP_Count.70.,

ALogP_Count.71., ALogP_Count.72., ALogP_Count.73., ALogP_Count.74.,

ALogP_Count.75., ALogP_Count.76., ALogP_Count.77., ALogP_Count.78.,

ALogP_Count.79., ALogP_Count.81., ALogP_Count.82., ALogP_Count.83.,

ALogP_Count.84., ALogP_Count.85., ALogP_Count.86., ALogP_Count.87.,

ALogP_Count.89., ALogP_Count.90., ALogP_Count.91., ALogP_Count.92.,

ALogP_Count.94., ALogP_Count.95., ALogP_Count.96., ALogP_Count.97.,

ALogP_Count.99., ALogP_Count.100., ALogP_Count.102., ALogP_Count.103.,

ALogP_Count.106., ALogP_Count.107., ALogP_Count.108., ALogP_Count.110.,

ALogP_Count.111., ALogP_Count.112., ALogP_Count.117., ALogP_Count.120.,

**FCFP_4**

FCFP_4.1., FCFP_4.2., FCFP_4.3., FCFP_4.4., FCFP_4.5., FCFP_4.6., FCFP_4.7.,

FCFP_4.8., FCFP_4.9., FCFP_4.10., FCFP_4.11., FCFP_4.12., FCFP_4.13., FCFP_4.14., FCFP_4.15., FCFP_4.16., FCFP_4.17., FCFP_4.18., FCFP_4.19., FCFP_4.20., FCFP_4.21., FCFP_4.22., FCFP_4.23., FCFP_4.24., FCFP_4.25., FCFP_4.26., FCFP_4.27., FCFP_4.28., FCFP_4.29., FCFP_4.30., FCFP_4.31., FCFP_4.32., FCFP_4.33., FCFP_4.34., FCFP_4.35., FCFP_4.36., FCFP_4.37., FCFP_4.38., FCFP_4.39., FCFP_4.40., FCFP_4.41., FCFP_4.42., FCFP_4.43., FCFP_4.44., FCFP_4.45., FCFP_4.46., FCFP_4.47., FCFP_4.48., FCFP_4.49., FCFP_4.50., FCFP_4.51., FCFP_4.52., FCFP_4.53., FCFP_4.54., FCFP_4.55., FCFP_4.56., FCFP_4.57., FCFP_4.58., FCFP_4.59., FCFP_4.60., FCFP_4.61., FCFP_4.62., FCFP_4.63., FCFP_4.64., FCFP_4.65., FCFP_4.66., FCFP_4.67., FCFP_4.68., FCFP_4.69., FCFP_4.70., FCFP_4.71., FCFP_4.72., FCFP_4.73., FCFP_4.74., FCFP_4.75., FCFP_4.76., FCFP_4.77., FCFP_4.78., FCFP_4.79., FCFP_4.80., FCFP_4.81., FCFP_4.82., FCFP_4.83., FCFP_4.84., FCFP_4.85., FCFP_4.86., FCFP_4.87., FCFP_4.88., FCFP_4.89., FCFP_4.90., FCFP_4.91., FCFP_4.92., FCFP_4.93., FCFP_4.94., FCFP_4.95., FCFP_4.96., FCFP_4.97., FCFP_4.98., FCFP_4.99., FCFP_4.100., FCFP_4.101., FCFP_4.102., FCFP_4.103.,FCFP_4.104., FCFP_4.105., FCFP_4.106., FCFP_4.107., FCFP_4.108., FCFP_4.109., FCFP_4.110., FCFP_4.111., FCFP_4.112., FCFP_4.113., FCFP_4.114., FCFP_4.115., FCFP_4.116., FCFP_4.117., FCFP_4.118., FCFP_4.119., FCFP_4.120., FCFP_4.121., FCFP_4.122., FCFP_4.123., FCFP_4.124., FCFP_4.125., FCFP_4.126., FCFP_4.127., FCFP_4.128., FCFP_4.129., FCFP_4.130., FCFP_4.131., FCFP_4.132., FCFP_4.133., FCFP_4.134., FCFP_4.135., FCFP_4.136., FCFP_4.137., FCFP_4.138., FCFP_4.139., FCFP_4.140., FCFP_4.141., FCFP_4.142., FCFP_4.143., FCFP_4.144., FCFP_4.145., FCFP_4.146., FCFP_4.147., FCFP_4.148., FCFP_4.149., FCFP_4.150., FCFP_4.151., FCFP_4.152., FCFP_4.153., FCFP_4.154., FCFP_4.155., FCFP_4.156., FCFP_4.157., FCFP_4.158., FCFP_4.159., FCFP_4.160., FCFP_4.161., FCFP_4.162., FCFP_4.163., FCFP_4.164., FCFP_4.165., FCFP_4.166., FCFP_4.167., FCFP_4.168., FCFP_4.169., FCFP_4.170., FCFP_4.171., FCFP_4.172., FCFP_4.173., FCFP_4.174., FCFP_4.175., FCFP_4.176., FCFP_4.177., FCFP_4.178., FCFP_4.179., FCFP_4.180., FCFP_4.181., FCFP_4.182., FCFP_4.183., FCFP_4.184., FCFP_4.185., FCFP_4.186., FCFP_4.187., FCFP_4.188., FCFP_4.189., FCFP_4.190., FCFP_4.191., FCFP_4.192., FCFP_4.193., FCFP_4.194., FCFP_4.195., FCFP_4.196., FCFP_4.197., FCFP_4.198., FCFP_4.199., FCFP_4.200., FCFP_4.201., FCFP_4.202., FCFP_4.203., FCFP_4.204., FCFP_4.205., FCFP_4.206., FCFP_4.207., FCFP_4.208., FCFP_4.209., FCFP_4.210., FCFP_4.211., FCFP_4.212., FCFP_4.213., FCFP_4.214., FCFP_4.215., FCFP_4.216., FCFP_4.217., FCFP_4.218., FCFP_4.219., FCFP_4.220., FCFP_4.221., FCFP_4.222., FCFP_4.223., FCFP_4.224., FCFP_4.225., FCFP_4.226., FCFP_4.227., FCFP_4.228., FCFP_4.229., FCFP_4.230., FCFP_4.231., FCFP_4.232., FCFP_4.233., FCFP_4.234., FCFP_4.235., FCFP_4.236., FCFP_4.237., FCFP_4.238., FCFP_4.239., FCFP_4.240., FCFP_4.241., FCFP_4.242., FCFP_4.243., FCFP_4.244., FCFP_4.245., FCFP_4.246., FCFP_4.247., FCFP_4.248., FCFP_4.249., FCFP_4.250., FCFP_4.251., FCFP_4.252., FCFP_4.253., FCFP_4.254., FCFP_4.255., FCFP_4.256.,

**ECFP_4**

ECFP_4.1., ECFP_4.2., ECFP_4.3., ECFP_4.4., ECFP_4.5., ECFP_4.6., ECFP_4.7., ECFP_4.8., ECFP_4.9., ECFP_4.10., ECFP_4.11., ECFP_4.12., ECFP_4.13., ECFP_4.14., ECFP_4.15., ECFP_4.16., ECFP_4.17., ECFP_4.18., ECFP_4.19., ECFP_4.20., ECFP_4.21., ECFP_4.22., ECFP_4.23., ECFP_4.24., ECFP_4.25., ECFP_4.26., ECFP_4.27., ECFP_4.28., ECFP_4.29., ECFP_4.30., ECFP_4.31., ECFP_4.32., ECFP_4.33., ECFP_4.34., ECFP_4.35., ECFP_4.36., ECFP_4.37., ECFP_4.38., ECFP_4.39., ECFP_4.40., ECFP_4.41., ECFP_4.42., ECFP_4.43., ECFP_4.44., ECFP_4.45., ECFP_4.46., ECFP_4.47., ECFP_4.48., ECFP_4.49., ECFP_4.50., ECFP_4.51., ECFP_4.52., ECFP_4.53., ECFP_4.54., ECFP_4.55., ECFP_4.56., ECFP_4.57., ECFP_4.58., ECFP_4.59., ECFP_4.60., ECFP_4.61., ECFP_4.62., ECFP_4.63., ECFP_4.64., ECFP_4.65., ECFP_4.66., ECFP_4.67., ECFP_4.68., ECFP_4.69., ECFP_4.70., ECFP_4.71., ECFP_4.72., ECFP_4.73., ECFP_4.74., ECFP_4.75., ECFP_4.76., ECFP_4.77., ECFP_4.78., ECFP_4.79., ECFP_4.80., ECFP_4.81., ECFP_4.82., ECFP_4.83., ECFP_4.84., ECFP_4.85., ECFP_4.86., ECFP_4.87., ECFP_4.88., ECFP_4.89., ECFP_4.90., ECFP_4.91., ECFP_4.92., ECFP_4.93., ECFP_4.94., ECFP_4.95., ECFP_4.96., ECFP_4.97., ECFP_4.98., ECFP_4.99., ECFP_4.100., ECFP_4.101., ECFP_4.102., ECFP_4.103., ECFP_4.104., ECFP_4.105., ECFP_4.106., ECFP_4.107., ECFP_4.108., ECFP_4.109., ECFP_4.110., ECFP_4.111., ECFP_4.112., ECFP_4.113., ECFP_4.114., ECFP_4.115., ECFP_4.116., ECFP_4.117., ECFP_4.118., ECFP_4.119., ECFP_4.120., ECFP_4.121., ECFP_4.122., ECFP_4.123., ECFP_4.124., ECFP_4.125., ECFP_4.126., ECFP_4.127., ECFP_4.128., ECFP_4.129., ECFP_4.130., ECFP_4.131., ECFP_4.132., ECFP_4.133., ECFP_4.134., ECFP_4.135., ECFP_4.136., ECFP_4.137., ECFP_4.138., ECFP_4.139., ECFP_4.140., ECFP_4.141., ECFP_4.142., ECFP_4.143., ECFP_4.144., ECFP_4.145., ECFP_4.146., ECFP_4.147., ECFP_4.148., ECFP_4.149., ECFP_4.150., ECFP_4.151., ECFP_4.152., ECFP_4.153., ECFP_4.154., ECFP_4.155., ECFP_4.156., ECFP_4.157., ECFP_4.158., ECFP_4.159., ECFP_4.160., ECFP_4.161., ECFP_4.162., ECFP_4.163., ECFP_4.164., ECFP_4.165., ECFP_4.166., ECFP_4.167., ECFP_4.168., ECFP_4.169., ECFP_4.170., ECFP_4.171., ECFP_4.172., ECFP_4.173., ECFP_4.174., ECFP_4.175., ECFP_4.176., ECFP_4.177., ECFP_4.178., ECFP_4.179., ECFP_4.180., ECFP_4.181., ECFP_4.182., ECFP_4.183., ECFP_4.184., ECFP_4.185., ECFP_4.186., ECFP_4.187., ECFP_4.188., ECFP_4.189., ECFP_4.190., ECFP_4.191., ECFP_4.192., ECFP_4.193., ECFP_4.194., ECFP_4.195., ECFP_4.196., ECFP_4.197., ECFP_4.198., ECFP_4.199., ECFP_4.200., ECFP_4.201., ECFP_4.202., ECFP_4.203., ECFP_4.204., ECFP_4.205., ECFP_4.206., ECFP_4.207., ECFP_4.208., ECFP_4.209., ECFP_4.210., ECFP_4.211., ECFP_4.212., ECFP_4.213., ECFP_4.214., ECFP_4.215., ECFP_4.216., ECFP_4.217., ECFP_4.218., ECFP_4.219., ECFP_4.220., ECFP_4.221., ECFP_4.222., ECFP_4.223., ECFP_4.224., ECFP_4.225., ECFP_4.226., ECFP_4.227., ECFP_4.228., ECFP_4.229., ECFP_4.230., ECFP_4.231., ECFP_4.232., ECFP_4.233., ECFP_4.234., ECFP_4.235., ECFP_4.236., ECFP_4.237., ECFP_4.238., ECFP_4.239., ECFP_4.240., ECFP_4.241., ECFP_4.242., ECFP_4.243., ECFP_4.244., ECFP_4.245., ECFP_4.246., ECFP_4.247., ECFP_4.248., ECFP_4.249., ECFP_4.250., ECFP_4.251., ECFP_4.252., ECFP_4.253., ECFP_4.254., ECFP_4.255., ECFP_4.256.

**Table** **S2. The calculation of** $\mathbf{Q}_{\boldsymbol{F}\boldsymbol{3}}^{\boldsymbol{2}}$**, RMSEC, RMSEP for RF, DNN, PLS, and MLR models with different training set.**

| **Training set numbers** | **RF** | | | **DNN** | | | **PLS** | | | **MLR** | | |
| --- | --- | --- | --- | --- | --- | --- | --- | --- | --- | --- | --- | --- |
|  | **RMSEC** | **RMSEP** | $\mathbf{Q}_{\boldsymbol{F}\boldsymbol{3}}^{\boldsymbol{2}}$ | **RMSEC** | **RMSEP** | $\mathbf{Q}_{\boldsymbol{F}\boldsymbol{3}}^{\boldsymbol{2}}$ | **RMSEC** | **RMSEP** | $\mathbf{Q}_{\boldsymbol{F}\boldsymbol{3}}^{\boldsymbol{2}}$ | **RMSEC** | **RMSEP** | $\mathbf{Q}_{\boldsymbol{F}\boldsymbol{3}}^{\boldsymbol{2}}$ |
| 6069 | 0.316 | 0.614 | 0.67 | 0.259 | 0.606 | 0.679 | 0.693 | 0.765 | 0.488 | 0.648 | 0.782 | 0.463 |
| 3035 | 0.342 | 0.678 | 0.607 | 0.255 | 0.714 | 0.567 | 1.085 | 0.829 | 0.414 | 0.648 | 0.852 | 0.381 |
| 303 | 0.413 | 0.957 | 0.157 | 0.249 | 0.947 | 0.181 | 4.836 | 1.073 | -0.05 | 0.261 | 2.938 | -6.88 |

**Table** **S3.1.** The top 84 compounds identify by RF models for TNBC test.

**Table S3.2.** The top 100 compounds identify by DNN models for TNBC test.

**Table S4.** The 63 compounds of the MOR agonist.

**Table S5.1.** The 40 compounds identify by RF models for MOR test.

**Table S5.2.** The 40 compounds identify by DNN models for MOR test.

**Figure S1. The IC50 calculation from the does-dependence test for TNBC cell-based assay.**


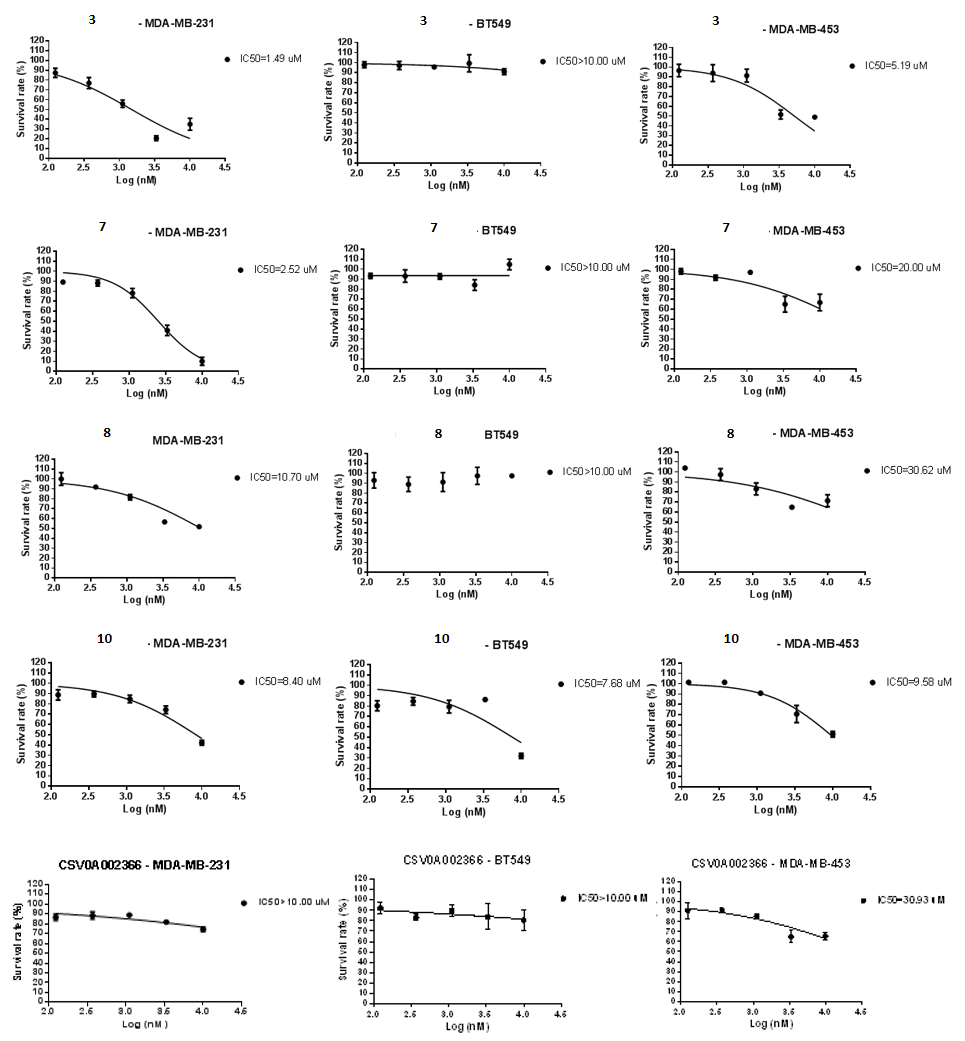


**Figure S2. The EC50 calculation from the does-dependence test for FLIPR calcium assay.**


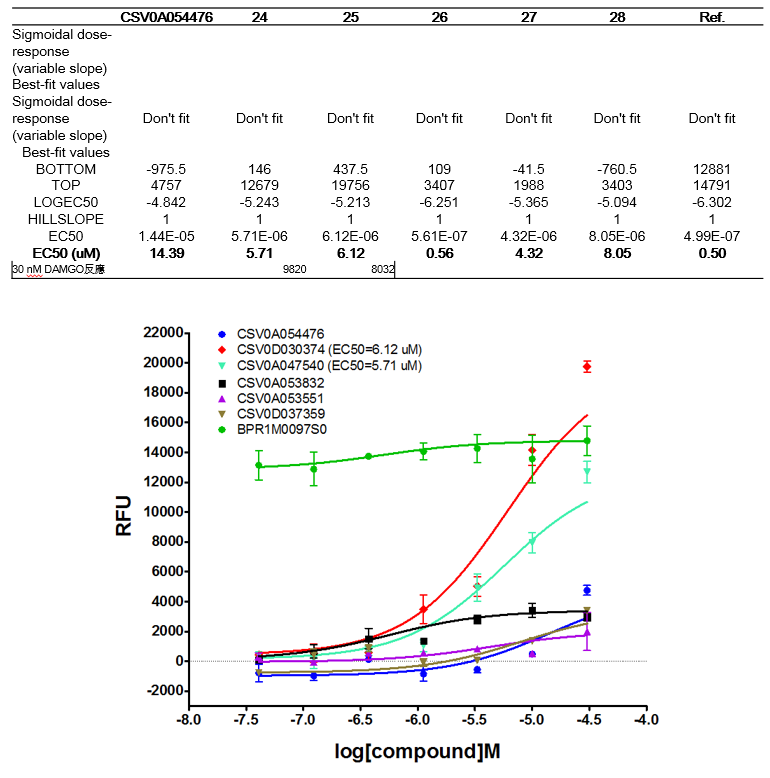


**The synthesis and identification of the 63 training set compounds** *N*-(1,2,3,4-tetrahydro-1-isoquinolinylmethyl)cyclohexanecarboxamide was reported in ref 1.

***N*-({2-[(4-Bromophenyl)sulfonyl]-1,2,3,4-tetrahydro-1-isoquinolinyl}methyl)cyclohexanecarboxamide (2)**, general procedure for compound **2**~**14**, **15**, **16**, **18**, **19**, **21**, **22**, **24**, and **25**.

To a solution of *N*-(1,2,3,4-tetrahydro-1-isoquinolinylmethyl)cyclohexanecarboxamide (0.06 g, 0.22 mmol) and *N*,*N*-diisopropylethylamine (0.03 mL, 0.15 mmol) in 0.4 mL of CH_2_Cl_2_ was added 4-bromobenzene-1-sulfonyl chloride (0.06 g, 0.22 mmol). The solution was stirred at room temperature for 2 h. To the solution was added 10 mL of water and extracted with CH_2_Cl_2_ (3 × 10 mL). The organic layer was dried over MgSO_4_ and the solvent was evaporated under reduced pressure. The crude product was purified by flash column chromatography (SiO_2_, CH_2_Cl_2_/MeOH = 98/2) to afford the sulfonamide as a white solid (0.10 g, 97%). Mp 74‒76 ^o^C; IR (neat) ν 3307 (-NH), 2929, 2852, 1648 (C=O), 1574, 1451, 1336 (sulfonamide), 1163 (sulfonamide), 1069, 751 cm^-1^; ^1^H NMR (300 MHz, CDCl_3_) δ 7.56 (d, *J* = 8.7 Hz, 2H, -ArH), 7.46 (d, *J* = 8.7 Hz, 2H, -ArH), 7.22‒7.08 (m, 3H, -ArH), 6.92 (d, *J* = 7.2 Hz, 1H, -ArH), 6.20 (br t, 1H, -NH), 5.04 (dd, *J* = 10.5, 2.4 Hz, 1H, -NCHCH_2_), 3.96‒3.84 (m, 1H, -NCH_2_CH_2_), 3.67 (ddd, *J* = 14.4. 6.3, 2.4 Hz, 1H, -NCH_2_CH), 3.57‒3.32 (m, 2H, -NCH_2_CH_2_, -NCH_2_CH), 2.62‒2.35 (m, 2H, -CH_2_CH_2_Ar), 2.16 (tt, *J* = 11.4, 2.1 1H, -CHC=O), 2.03‒1.58 (m, overlapped with br s at 1.69, 5H, -cyclohexyl), 1.48 (q, *J* = 2.0 Hz, 2H, -cyclohexyl), 1.38‒1.13 (m, 3H, -cyclohexyl); ^13^C NMR (100 MHz, CDCl_3_) δ 177.1, 139.8, 132.9, 132.6, 132.5, 129.3, 128.5, 127.8, 127.8, 127.4, 126.9, 56.6, 45.6, 44.1, 39.2, 29.8, 29.7, 26.4, 26.0; MS (ESI) m/z 491.3 (M+H), 415.8, 308.7, 301.7, 273.7; HRMS (ESI) calcd. for C_23_H_27_BrN_2_O_3_S (M+H) 491.1004, found 491.1000.

General procedure for compound **17**, **20**, **23**, and **63**.

The synthesis and identification of 2-(1,2,3,4-tetrahydro-1-isoquinolinylmethyl)-1*H*-isoindole-1,3(2*H*)-dione was reported in ref 1.

**2-[(2-{[4-Bromo-2-(trifluoromethoxy)phenyl]sulfonyl}-1,2,3,4-tetrahydro-1-isoquinolinyl)methyl]-1*H*-isoindole-1,3(2*H*)-dione (S1)**

The reaction of 2-(1,2,3,4-tetrahydro-1-isoquinolinylmethyl)-1*H*-isoindole-1,3(2*H*)-dione (0.04 g, 0.15 mmol) with 4-bromo-2-(trifluoromethoxy)benzene-1-sulfonyl chloride (0.03 mL, 0.16 mmol) was followed the general procedure B. The crude product was purified by flash column chromatography (SiO_2_, ethyl acetate/hexane = 1/3) to afford the sulfonamide as a white solid (0.06 g, 63%).Mp 193‒195 ^o^C; IR (neat) ν 3026, 2923, 1774, 1716, 1580, 1397, 1351 (sulfonamide), 1248, 1210, 1167 (sulfonamide), 1069, 942, 749 cm^-1^; ^1^H NMR (300 MHz, CDCl_3_) δ 7.82‒7.72 (m, 5H, -ArH), 7.32‒7.27 (m, 2H, -ArH), 7.26‒7.20 (m, 2H, -ArH) 6.97 (s, 1H, -ArH), 5.37 (dd, *J* = 11.1, 3.6 Hz, 1H,- NCHCH_2_), 4.14‒4.01 (m, 2H, -NCH_2_CH_2_, -NCH_2_CH), 3.86‒3.73 (m, 2H, -NCH_2_CH_2_, -NCH_2_CH), 2.81‒2.76 (m, 2H, -CH_2_CH_2_Ar); ^13^C NMR (100 MHz, CDCl_3_) δ 168.1, 145.9, 134.3, 133.5, 132.7, 132.2, 132.0, 131.8, 129.8, 129.4, 127.9, 127.3, 126.8, 123.5, 122.6, 120.0 (q, *J*_C-F_ = 260.8 Hz), 55.2, 41.6, 39.1, 29.9, 27.8; MS (ESI) m/z 595.0 (M+H), 215.1, 144.9; HRMS (ESI) calcd. for C_25_H_18_BrF_3_N_2_O_5_S (M+H) 595.0150, found 595.0145

**1-(2-{[4-Bromo-2-(trifluoromethoxy)phenyl]sulfonyl}-1,2,3,4-tetrahydro-1-isoquinolinyl)methanamine (62)**

To a solution of 2-[(2-{[4-bromo-2-(trifluoromethoxy)phenyl]sulfonyl}-1,2,3,4-tetrahydro-1-isoquinolinyl)methyl]-1H-isoindole-1,3(2*H*)-dione **S1** ( 2.35 g, 3.95 mmol) in ethanol/CHCl_3_ (10.2/24.1 mL) was added hydrazine. The solution was refluxed for 72 h, cooled to room temperature and concentrated under reduced pressure. The residue was dissolved in 100 mL of CH_2_Cl_2_ and filtered. The filtrate was added 50 mL of water and extracted with CH_2_Cl_2_ (2 × 100 mL).The organic layer was dried over MgSO_4_ and the solvent was evaporated under reduced pressure to afford the amine as a brown solid (1.89 g, 100%). Mp 87‒88 ^o^C; IR (neat) ν 3380 (-NH_2_), 2927, 2853, 1580, 1394, 1339 (sulfonamide), 1249, 1210, 1168 (sulfonamide), 1071, 1023, 942, 745, 606 cm^-1^; ^1^H NMR (300 MHz, CDCl_3_) δ 7.95 (d, *J* = 8.4 Hz, 1H, -ArH), 7.49 (d, *J* = 8.4 Hz, 1H,-ArH), 7.35 (s, 1H, -ArH), 7.20‒7.07 (m, 3H, -ArH), 6.99 (d, *J* = 8.4 Hz, 1H, -ArH), 4.96 (dd, *J* = 9.0, 4.8 Hz, 1H, -NCHCH_2_), 3.86 (ddd, *J* = 14.4, 6.0, 2.7 Hz, 1H, -NCH_2_CH_2_), 3.51 (ddd, *J* = 14.4, 11.4, 5.1 Hz, 1H, -NCH_2_CH_2_), 3.04‒2.90 (m, 2H, -NCH_2_CH), 2.63‒2.49 (m, 2H, -CH_2_CH_2_Ar); ^13^C NMR (100 MHz, CDCl_3_) δ 146.3, 134.0, 132.8, 132.8, 132.0, 129.7, 129.2, 128.2, 127.3, 127.2, 126.7, 123.2, 120.0 (q, *J*_C-F_ = 261.1 Hz), 60.4, 47.7, 39.3, 27.2; MS (ESI) m/z 465.0 (M+H); HRMS (ESI) calcd. for C_17_H_16_BrF_3_N_2_O_3_S (M+H) 465.0095, found 465.0090

***N*-[(2-{[4-Bromo-2-(trifluoromethoxy)phenyl]sulfonyl}-1,2,3,4-tetrahydro-1-isoquinolinyl)methyl]-1-piperidinecarboxamide (17)**

The reaction of 1-(2-{[4-bromo-2-(trifluoromethoxy)phenyl]sulfonyl}-1,2,3,4-tetrahydro-1-isoquinolinyl)methanamine **62** (0.07 g, 0.15 mmol) with 1-piperidinecarbonyl chloride (0.02 mL, 0.18 mmol) and triethylamine (0.04 mL, 0.30 mmol) was followed the general procedure C. The crude product was purified by flash column chromatography (SiO_2_, CH_2_Cl_2_/MeOH = 97/3) to afford the amide as a yellow solid (0.08 g, 89%). Mp 65‒67 ^o^C; IR (neat) ν 3352, 2937, 2855, 1629 (C=O), 1580 (C=O), 1531 (C=O), 1394, 1339, 1249, 1211, 1165, 1084, 1023, 942, 747 cm^-1^; ^1^H NMR (300 MHz, (CD_3_)_2_CO) δ 7.96 (d, *J* = 8.4 Hz, 1H, -ArH), 7.73 (d, *J* = 8.4 Hz, 1H, -ArH), 7.57 (s, 1H, -ArH), 7.23‒7.13 (m, 3H, -ArH), 7.05 (d, *J* = 7.2 Hz, 1H, -ArH), 5.95 (br t, 1H, -NH), 5.20 (t, *J* = 7.2 Hz, 1H, -NCHCH_2_), 3.99 (dd, *J* = 15.6, 5.7 Hz, 1H, -NCH_2_CH_2_), 3.66 (ddd, *J* = 15.6, 11.7, 5.7 Hz, 1H, -NCH_2_CH_2_), 3.44 (t, *J* = 7.2 Hz, 2H, -NCH_2_CH), 3.34‒3.20 (m, 4H, -CH_2_NCH_2_), 2.75‒2.54 (m, 2H, -CH_2_CH_2_Ar), 1.62‒1.42 (m, 6H, -piperidinyl); ^13^C NMR (75 MHz, CDCl_3_) δ 157.7, 146.2, 133.4, 132.8, 132.6, 131.9, 129.8, 129.2, 128.2, 127.5, 127.4, 126.7, 123.2, 120.0 (q, *J*_C-F_ = 261.1 Hz), 57.2, 45.5, 45.0, 39.0, 27.2, 25.8, 24.7; MS (ESI) m/z 576.0 (M+H), 207.1, 197.2, 185.2; HRMS (ESI) calcd. for C_23_H_25_BrF_3_N_3_O_4_S (M+H) 576.0779, found 576.0774.

The synthetic procedures and identifications of compound **26**~**56**, and **58**~**61** were reported in ref 1.

The synthesis of TNBC inhibitors.


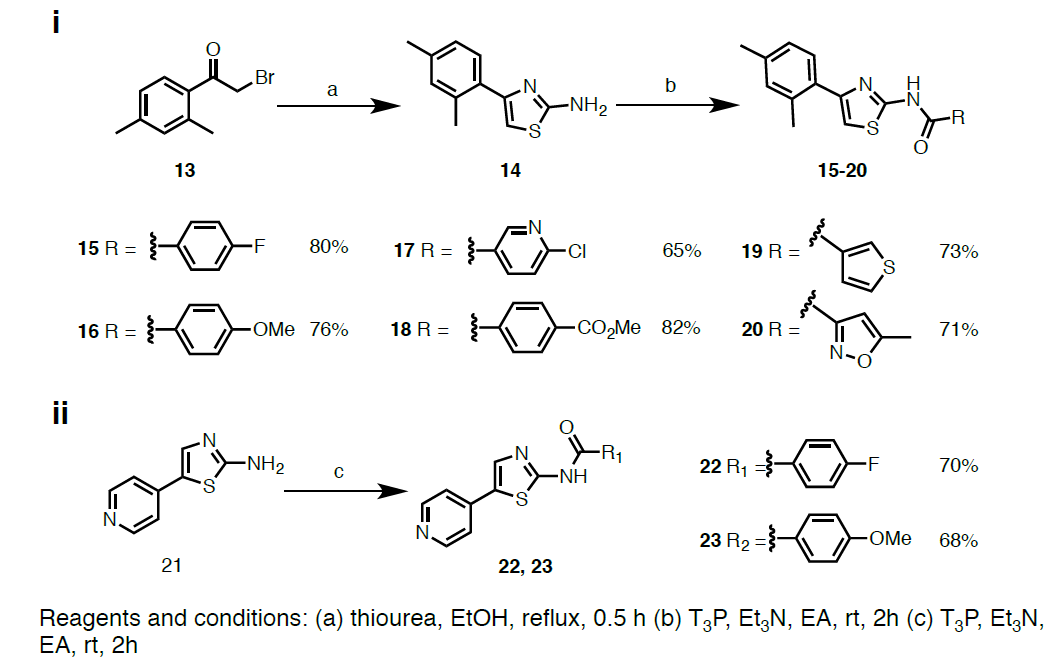


**4-(2,4-dimethylphenyl)thiazol-2-amine (14)** 4-(2,4-Dimethyl-phenyl)thiazol-2-ylamine (**13**). The mixture of 2-bromo-1-(2,4-dimethylphenyl)ethanone (2.56 g, 11.3 mmol) and thiourea (900 mg, 511.8 mmol) in anhydrous EtOH (40 mL) was heated to reflux for 30 min. After that, the solvent was removed in vacuo and the saturated aqueous NaHCO_3_ was added to make the mixture basic (pH = 8-9). Then the mixture was extracted with CH_2_Cl_2_ (3 × 60 mL). The combined organic phases were dried with anhydrous MgSO_4._ After removal of all the solvent, the residue was purified by silica gel chromatography EtOAc/hexane (1:2) to afford product **14** (2.06 g, 89%) as a solid. ^1^H NMR (400 MHz, CDCl_3_) δ 7.43 (d, *J* = 7.6 Hz, 1H), 7.05-7.01 (m, 2H), 6.43 (s, 1H), 5.02 (br, 2H), 2.41 (s, 3H), 2.33 (s, 3H). LCMS (ESI) m/z: 205.1 [M + H]^+^.

**N-(4-(2,4-dimethylphenyl)thiazol-2-yl)-4-fluorobenzamide** (**15**) A mixture of compound **14** (500 mg; 2.45 mmol), Et_3_N (1.3 mL), 4-fluorobenzoic acid (412 mg; 9.7 mmol), and T_3_P (312 mg; 4.9 mmol) were in ethyl acetate was stirred in a glass vessel for 2 hours at room temperature. Then the reaction mixture was poured into water and the aqueous layer was extracted with ethyl acetate. The combined organic extracts were washed with water and brine, dried over anhydrous Na_2_SO_4_, filtered, and concentrated under reduced pressure to give a crude residue, which was purified by column chromatography with DCM/hexane (20:80) to afford compound **15**. Yield: 80 %; ^1^H NMR (400 MHz, CDCl_3_) *δ* 7.78-7.74 (m, 2H), 7.28-7.26 (m, 1H), 7.02 (t, *J* = 8.8 Hz, 2H), 6.93-6.91 (m, 3H), 2.32 (s, 3H), 2.28 (s, 3H); ^13^C NMR (100 MHz, CDCl_3_) *δ* 165.21 (*J_C-F_* = 253.0 Hz), 164.98, 159.40, 150.07, 136.74, 131.57, 131.15, 130.15 ( *J_C-F_* = 9.2 Hz), 129.58, 128.35 ( *J_C-F_* = 3.1 Hz), 126.61 (s), 115.50 ( *J_C-F_* = 22.2 Hz ), 110.77, 21.12, 20.71; LCMS (ESI) m/z: 327.1 [M + H]^+^; HRMS (ESI) calcd for C_18_H_15_FN_2_OS [M + H]^+^ m/z: 327.0967; found: 327.0967; HPLC purity = 99.9%, *t*R = 42.75 min.

**N-(4-(2,4-dimethylphenyl)thiazol-2-yl)-4-methoxybenzamide (16)** Following the similar reaction and workup procedures for **15,** the crude residue was purified by silica gel column chromatography with EtOAc/hexane (10:90) to give **16**. Yield: 76 %; ^1^H NMR (300 MHz, CDCl_3_) *δ* 7.92 (d, *J* = 8.7 Hz, 2H), 7.45 (d, *J* = 7.5 Hz, 1H), 7.05 (m, 4H), 6.91(s, 1H) 3.89 (s, 3H), 2.42 (s, 3H), 2.36 (s, 3H); ^13^C NMR (100 MHz, CDCl_3_) *δ* 165.18, 162.97, 159.02, 150.13, 137.82, 135.67, 131.61, 131.54, 129.64, 126.61, 124.41, 113.81, 110.53, 55.56, 29.90, 21.20, 20.91; LCMS (ESI) m/z: 339.3 [M + H]^+^; HRMS (ESI) calcd for C_19_H_18_N_2_O_2_S [M + H]^+^ m/z: 339.1167; found: 339.1161; HPLC purity = 97.4%, *t*R = 42.11 min.

**6-chloro-N-(4-(2,4-dimethylphenyl)thiazol-2-yl)nicotinamide** (**17**) Following the similar reaction and workup procedures for **15,** the crude residue was purified by silica gel column chromatography with EtOAc/hexane (10:90) to give **17**. Yield: 65 %; ^1^H NMR (300 MHz, CDCl_3_) *δ* 8.56 (d, *J* = 2.4 Hz, 1H), 7.77 (dd, *J* = 8.4, 2.4 Hz, 1H), 7.12-7.08 (m, 2H), 6.94 (s, 1H), 6.80-6.77 (m, 2H); ^13^C NMR (100 MHz, CDCl_3_) *δ* 163.59, 159.62, 154.89, 149.64, 149.09, 138.34, 137.60, 135.67, 131.49, 130.74, 129.64, 126.97, 126.55, 123.82, 111.34, 21.11, 20.52; LCMS (ESI) m/z: 344.1 [M + H]^+^ ; HRMS (ESI) calcd for C_17_H_14_ClN_3_OS [M + H]^+^ m/z: 344.0624; found: 344.0626; HPLC purity = 98.7%, *t*R = 40.17 min.

**methyl 4-((4-(2,4-dimethylphenyl)thiazol-2-yl)carbamoyl)benzoate (18)** Following the similar reaction and workup procedures for **15,** the crude residue was purified by silica gel column chromatography with EtOAc/hexane (10:90) to give **18**. Yield: 82%; ^1^H NMR (400 MHz, CDCl_3_) *δ* 8.01 (d, *J* = 8.8 Hz, 2H), 7.80 (d, *J* = 8.8 Hz, 2H), 7.26-7.24 (m, 1H), 6.93 (s, 1H), 6.89-6.87 (m, 2H), 3.97 (s, 3H), 2.30 (s, 3H), 2.26 (s, 3H); ^13^C NMR (100 MHz, CDCl_3_) *δ* 166.30, 165.14, 159.05, 150.11, 137.97, 135.93, 135.50, 133.39, 131.61, 131.09, 129.64, 129.54, 127.65, 126.66 , 110.96, 52.66, 21.12, 20.78; LCMS (ESI) m/z: 367.3 [M + H]^+^; HRMS (ESI) calcd for C_20_H_18_N_2_O_3_S [M + H]^+^ m/z: 367.1116; found: 367.1124; HPLC purity = 99.1%, *t*R = 42.91 min.

**N-(4-(2,4-dimethylphenyl)thiazol-2-yl)thiophene-2-carboxamide (19)** Following the similar reaction and workup procedures for **15,** the crude residue was purified by silica gel column chromatography with EtOAc/hexane (10:90) to give **19**. Yield: 73 %; ^1^H NMR (300 MHz, CDCl_3_) *δ* 7.90 (dd, *J* = 3.0, 1.5 Hz, 1H), 7.40 (dd, *J* = 5.1, 1.5 Hz, 1H), 7.35 (d, *J* = 7.5 Hz, 1H), 7.30 (dd, *J* =5.1, 3.0 Hz, 1H), 7.05-6.95 (m, 2H), 6.92 (s, 1H), 2.37 (s, 3H), 2.30 (s, 3H); ^13^C NMR (100 MHz, CDCl_3_) *δ* 160.84, 158.61, 150.29, 138.07, 135.80, 135.17, 131.72, 131.50, 130.42, 129.67, 126.77, 126.65, 126.46, 110.81, 21.23, 20.90; LCMS (ESI) m/z: 315.1 [M + H]^+^; HRMS (ESI) calcd for C_16_H_14_N_2_OS_2_ [M + H]^+^ m/z: 315.0626; found: 315.0625; HPLC purity = 99.1%, *t*R = 40.55 min.

**N-(4-(2,4-dimethylphenyl)thiazol-2-yl)-5-methylisoxazole-3-carboxamide (20)** Following the similar reaction and workup procedures for **15,** the crude residue was purified by silica gel column chromatography with EtOAc/hexane (10:90) to give **20**. Yield: 71 %; ^1^H NMR (400 MHz, CDCl_3_) *δ* 7.46 (d, *J* = 7.5 Hz, 1H), 7.08-7.05 (m, 2H), 6.95 (s, 1H), 6.57 (s, 1H), 2.54 (s, 3H), 2.43 (s, 3H), 2.35 (s, 3H); ^13^C NMR (100 MHz, CDCl_3_) *δ* 172.22, 157.44, 156.80, 155.66, 150.85, 138.02, 136.00, 131.75, 131.64, 129.74, 126.73, 111.00, 101.59, 21.30, 21.18, 12.60; LCMS (ESI) m/z: 314.2 [M + H]^+^; HRMS (ESI) calcd for C_16_H_15_N_3_O_2_S [M + H]^+^ m/z: 314.0963; found: 314.0955; HPLC purity = 100%, *t*R = 39.87 min

**4-fluoro-N-(5-(pyridin-4-yl)thiazol-2-yl)benzamide (22)** Following the similar reaction and workup procedures for **15,** the crude residue was purified by silica gel column chromatography with DCM/MeOH (98:2) to give **22**. Yield: 70 %; ^1^H NMR (400 MHz, DMSO-*d*_6_) *δ* 8.57 (dd, *J* = 4.4 Hz, 1.6 Hz, 2H), 8.29 (s, 1H), 8.22-8.18 (m, 2H), 7.65 (dd, *J* = 4.4 Hz, 1.6 Hz, 2H), 7.43-7.38 (m, 2H); ^13^C NMR (100 MHz, DMSO-*d*_6_) *δ* 164.74 ( *J_C-F_* = 250.8 Hz ), 164.41, 159.74, 150.31, 138.89, 137.09, 131.15 ( *J_C-F_* = 9.3 Hz ), 128.50 ( *J_C-F_* = 2.3 Hz ), 128.39, 119.75, 115.66 ( *J_C-F_* = 22.2 Hz ); LCMS (ESI) m/z: 300.0 [M + H]^+^; HRMS (ESI) calcd for C_15_H_10_FN_3_OS [M + H]^+^ m/z: 300.0607; found: 300.0606; HPLC purity = 100%, *t*R = 17.54 min

**4-methoxy-N-(5-(pyridin-4-yl)thiazol-2-yl)benzamide (23)** Following the similar reaction and workup procedures for **15,** the crude residue was purified by silica gel column chromatography with EtOAc/hexane (30:70) to give **23**. Yield: 68%; ^1^H NMR (400 MHz, DMSO-*d*_6_) *δ* 8.56 (d, *J* = 6.0 Hz, 2H), 8.27 (s, 1H), 8.13 (d, *J* = 9.2 Hz, 2H), 7.64 (d, *J* = 6.0 Hz, 2H) 7.09 (d, *J* = 9.2 Hz, 2H), 3.86 (s, 3H); ^13^C NMR (100 MHz, DMSO-*d*_6_) *δ* 164.59, 162.89, 159.73, 150.24, 139.07, 137.28, 130.38, 128.27, 123.75, 119.78, 113.96, 55.56; LCMS (ESI) m/z: 312.2 [M + H]^+^; HRMS (ESI) calcd for C_16_H_13_N_3_O_2_S [M + H]^+^ m/z: 312.0807; found: 312.0805; HPLC purity = 96.4%, *t*R = 17.39 min.

***
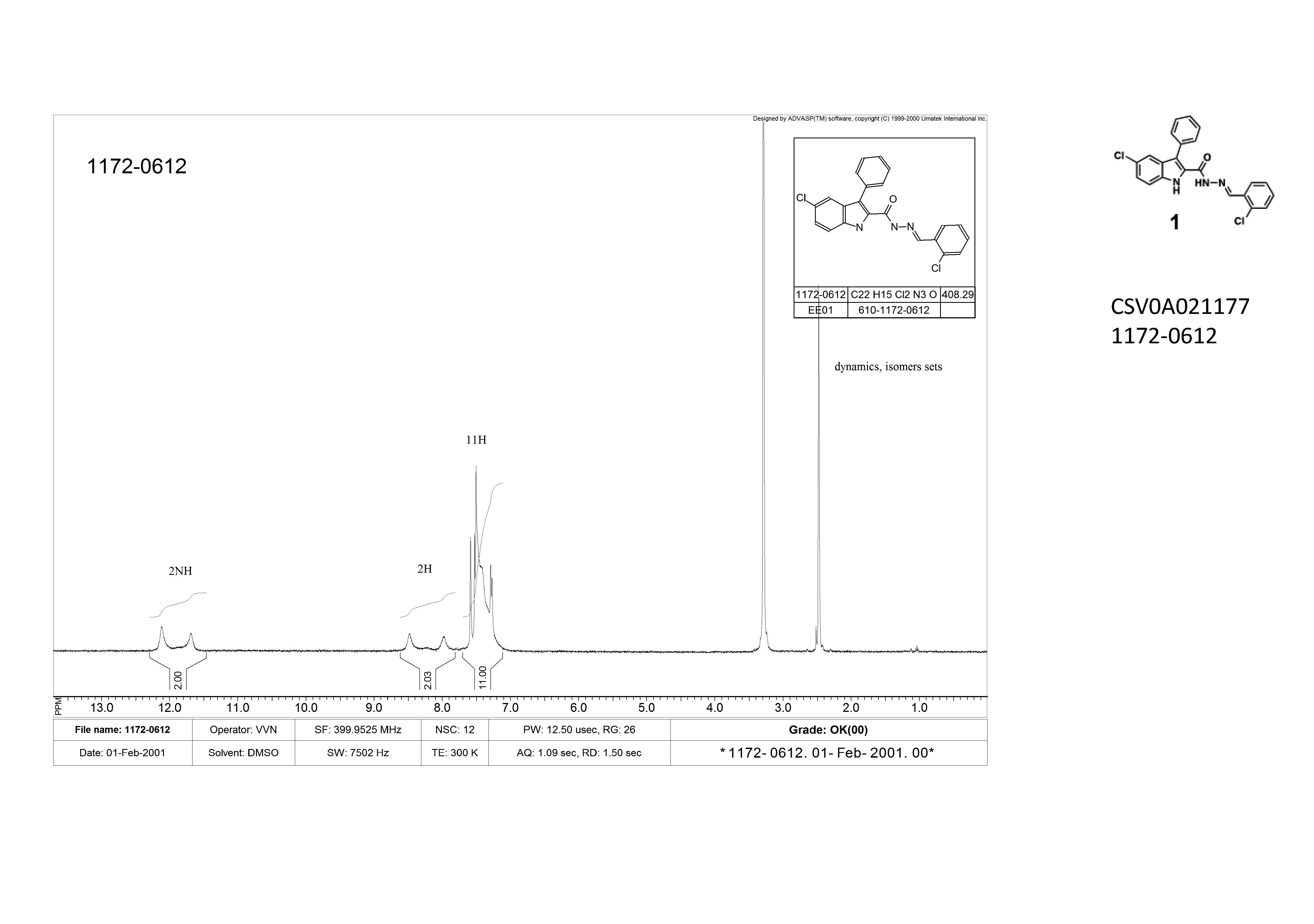
***


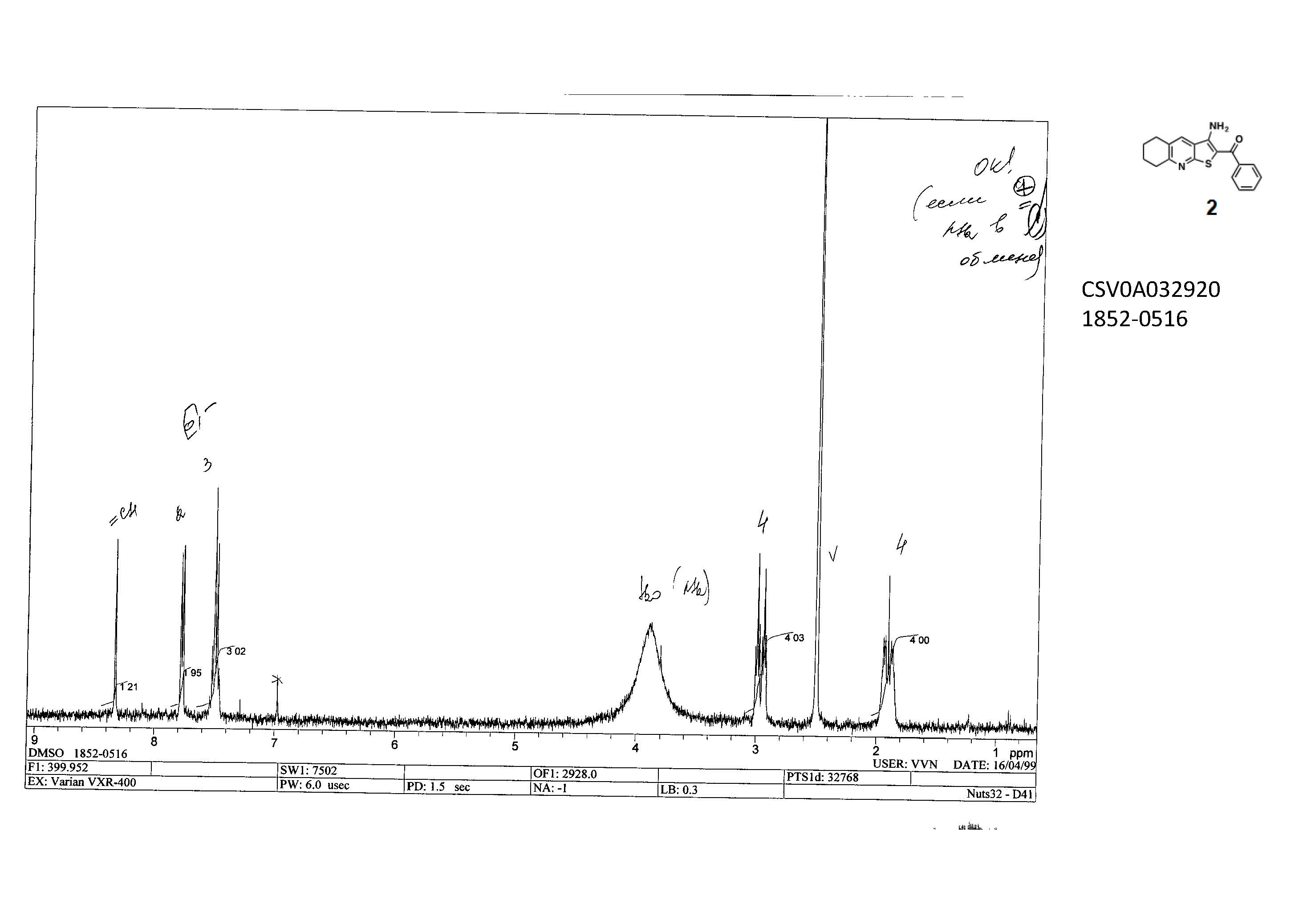


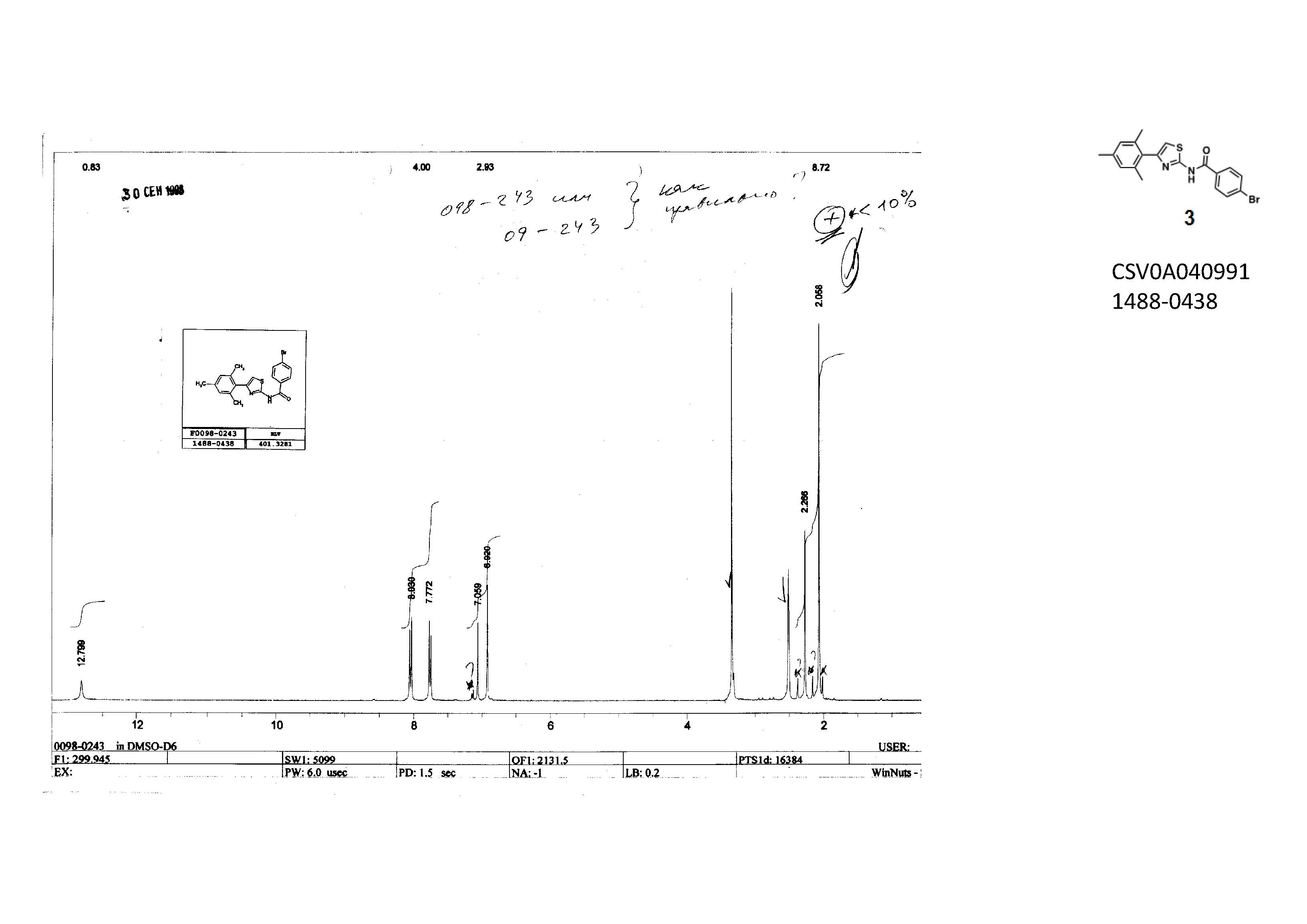


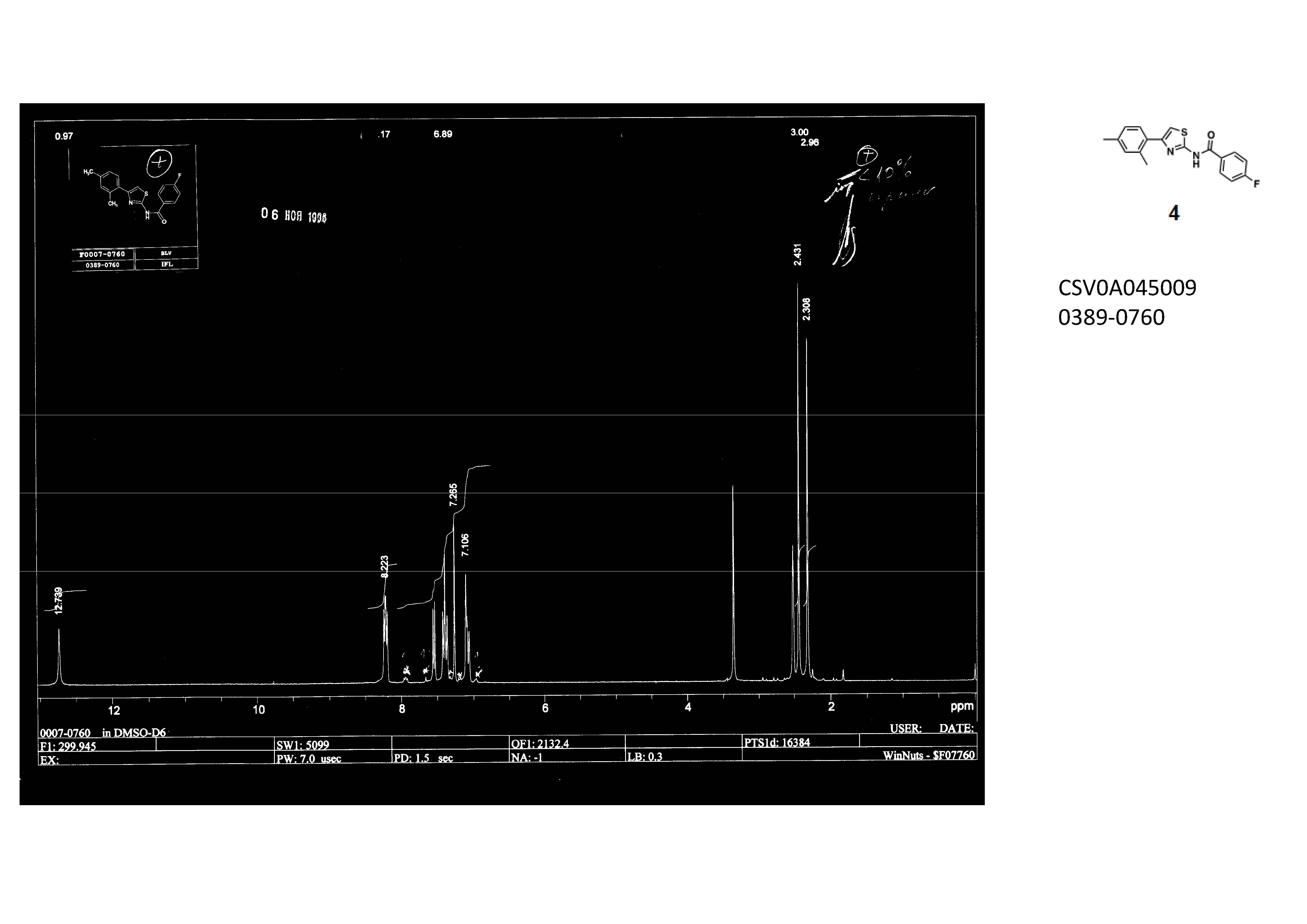


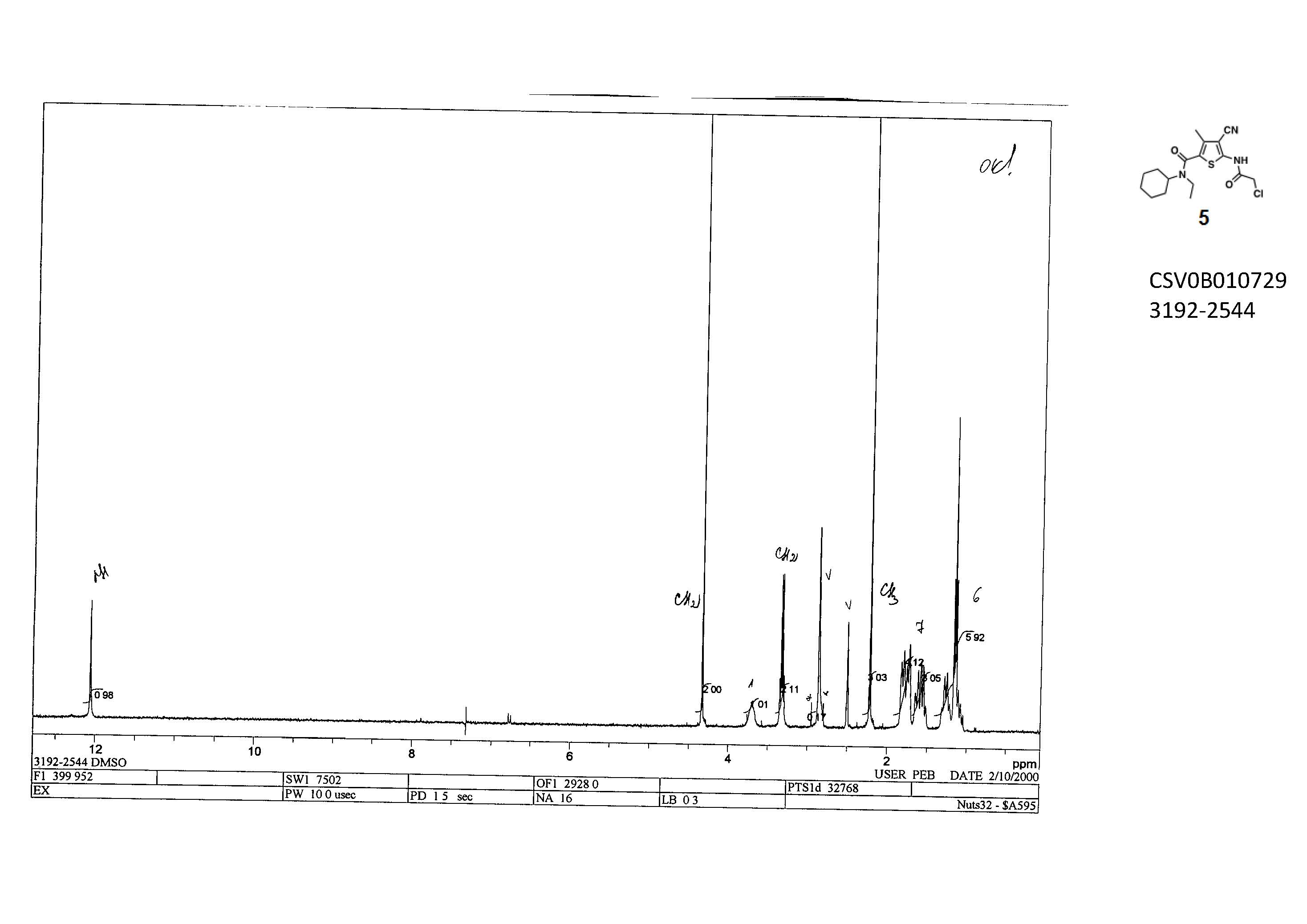


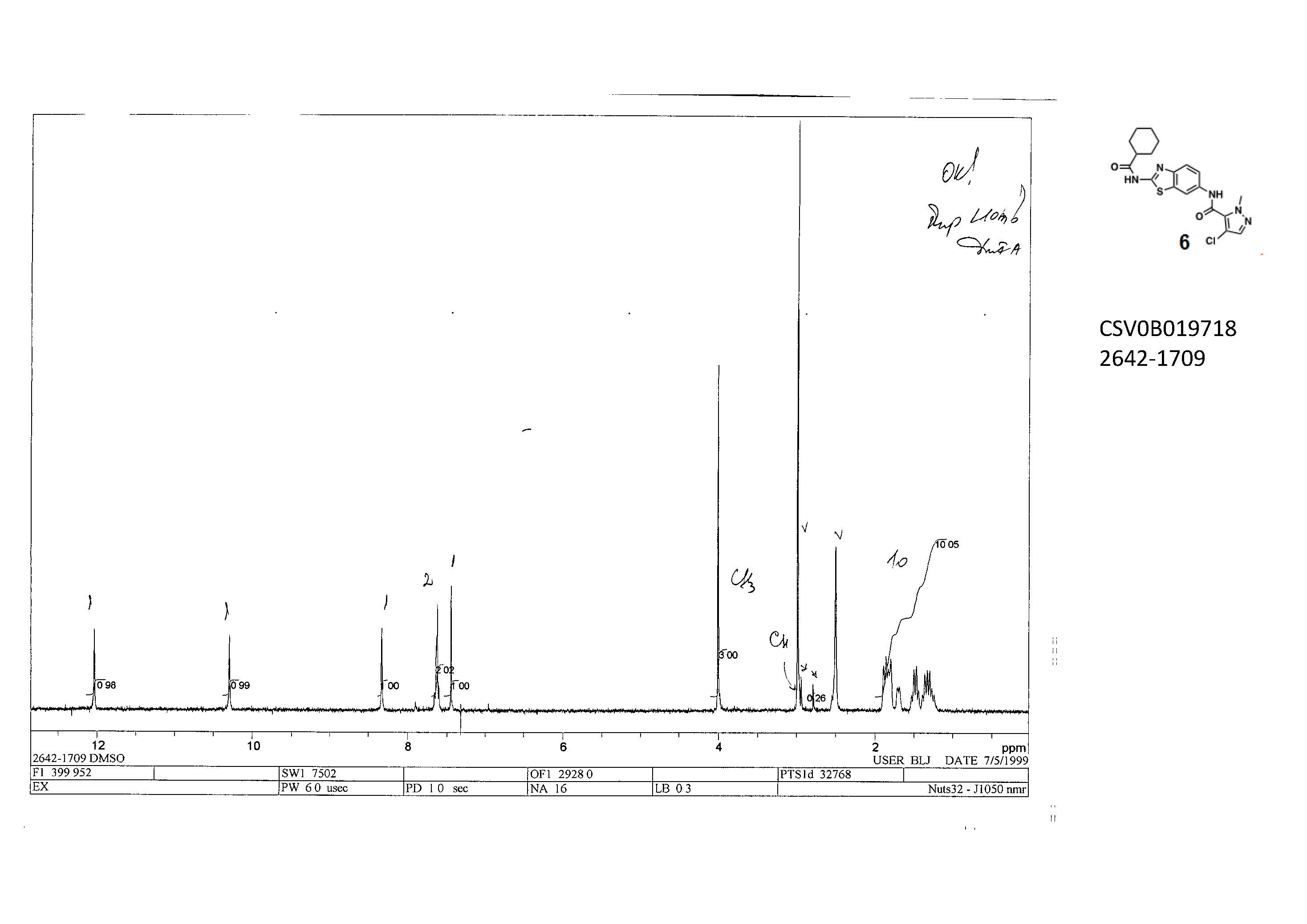


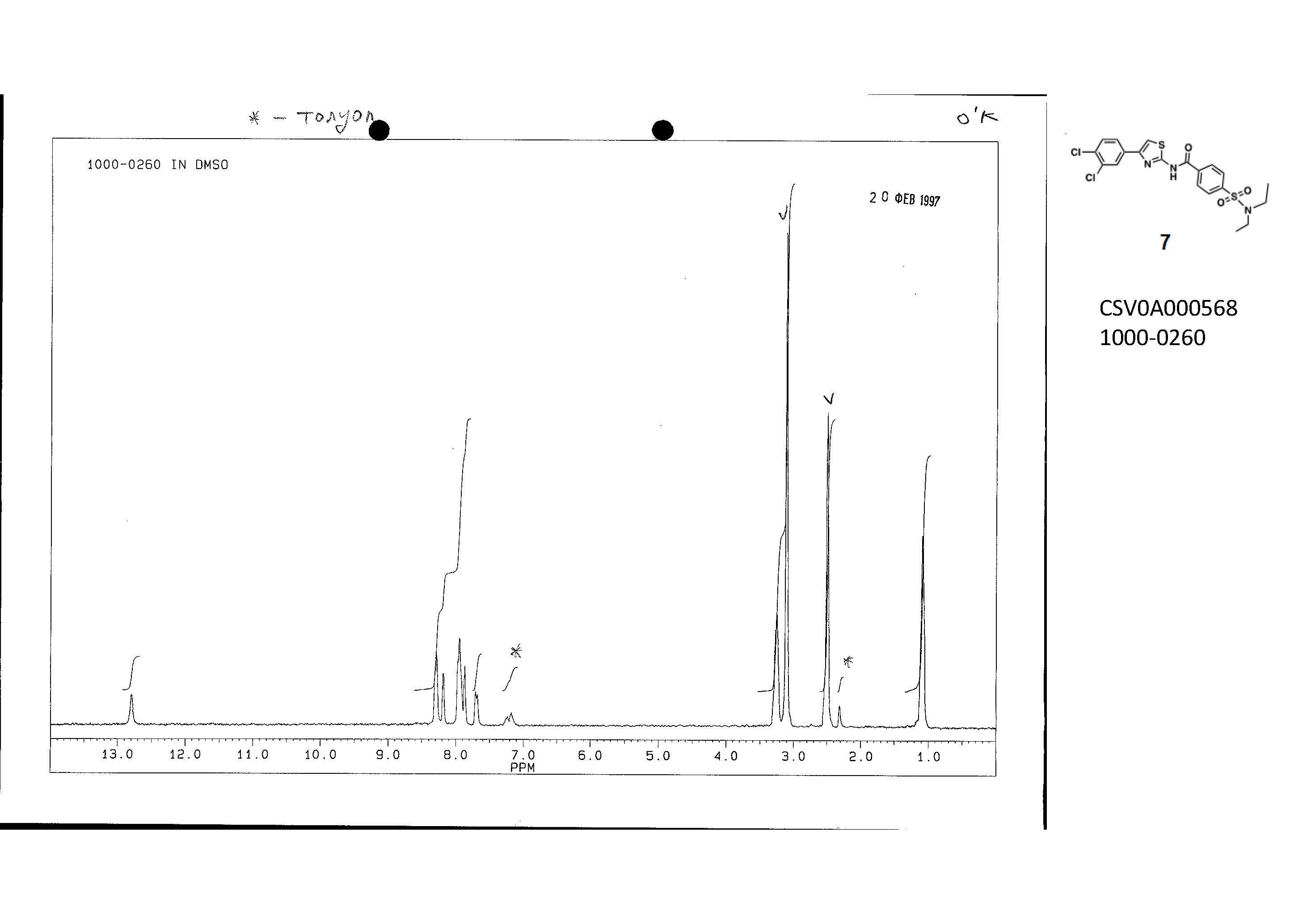


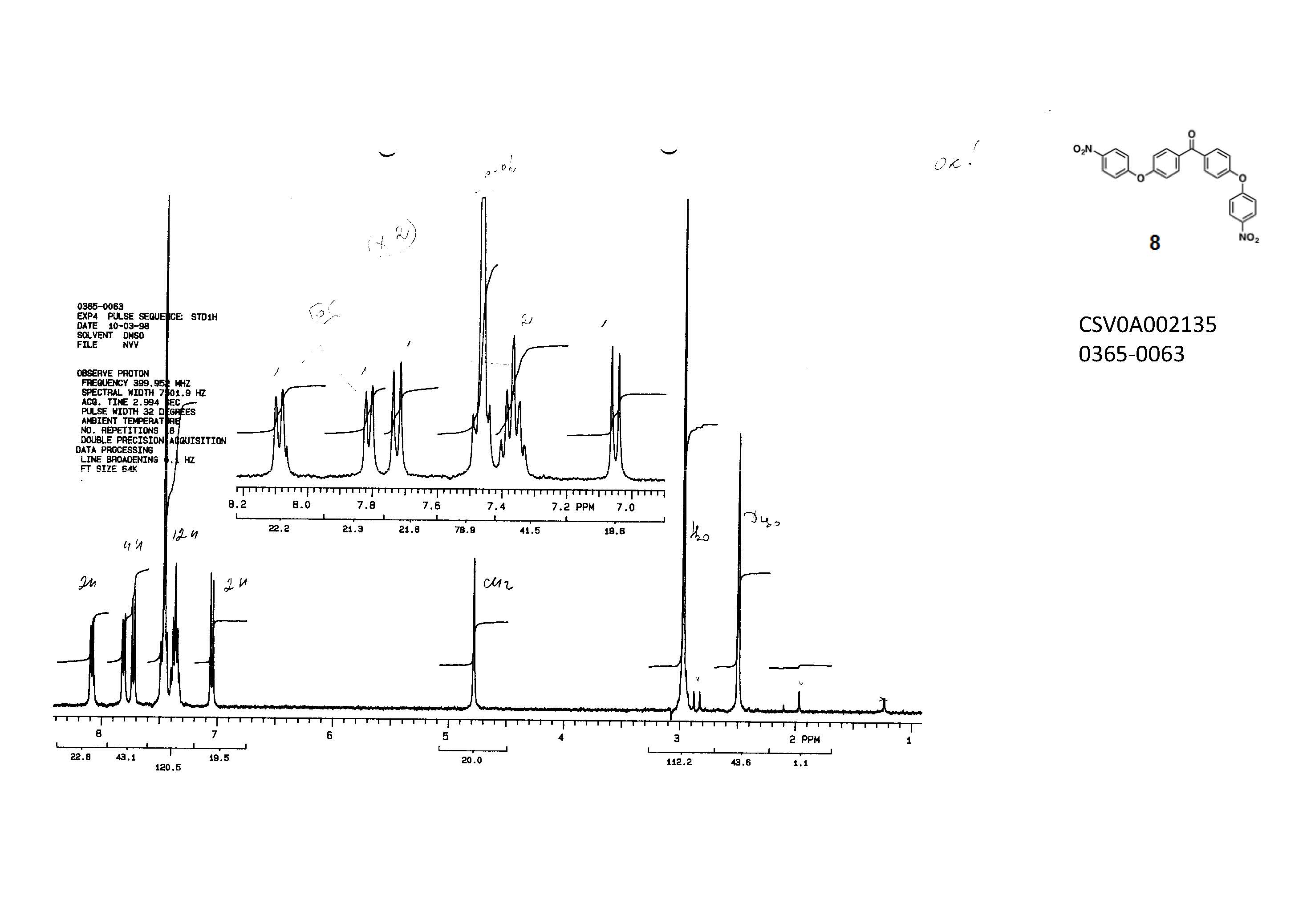


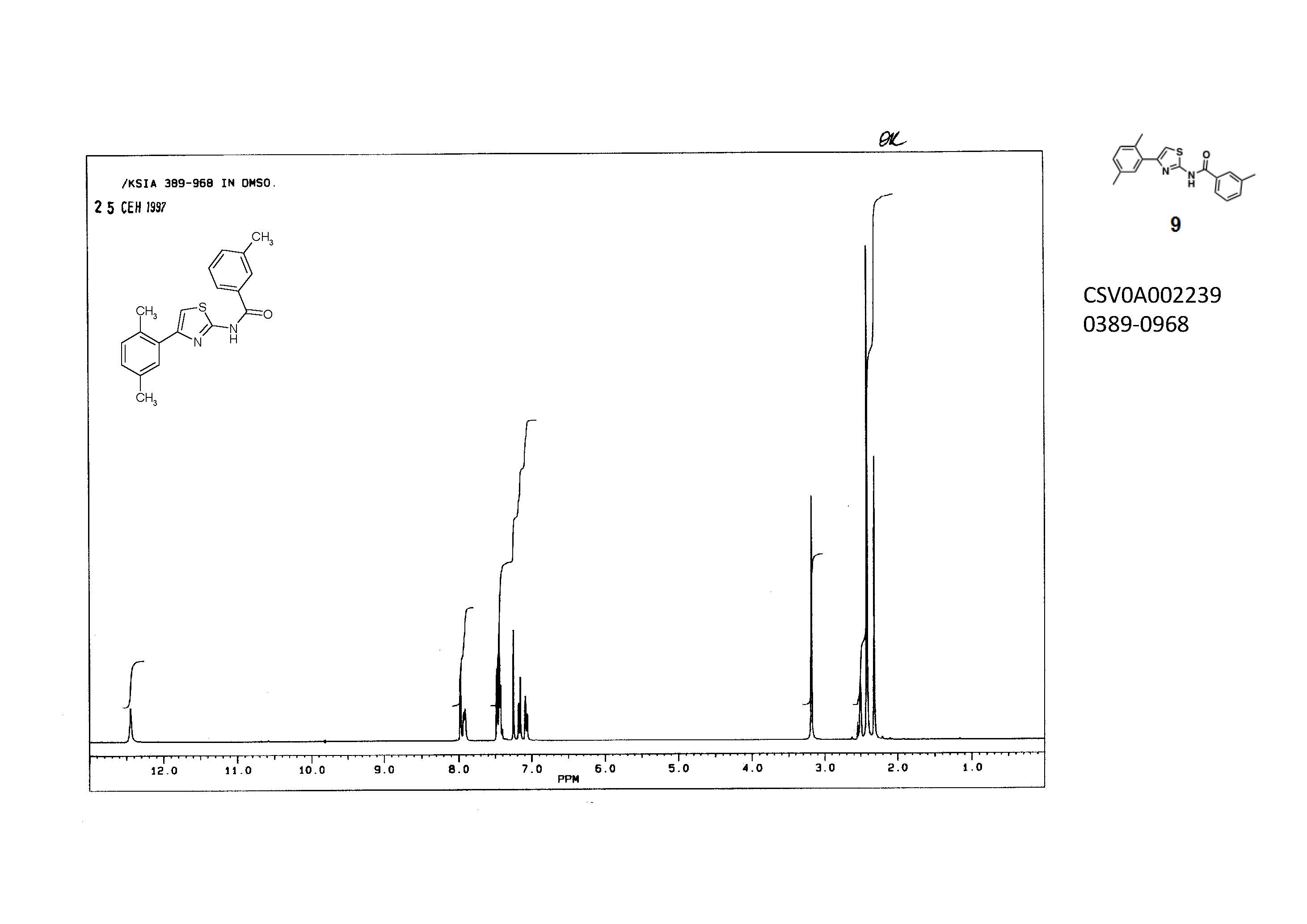


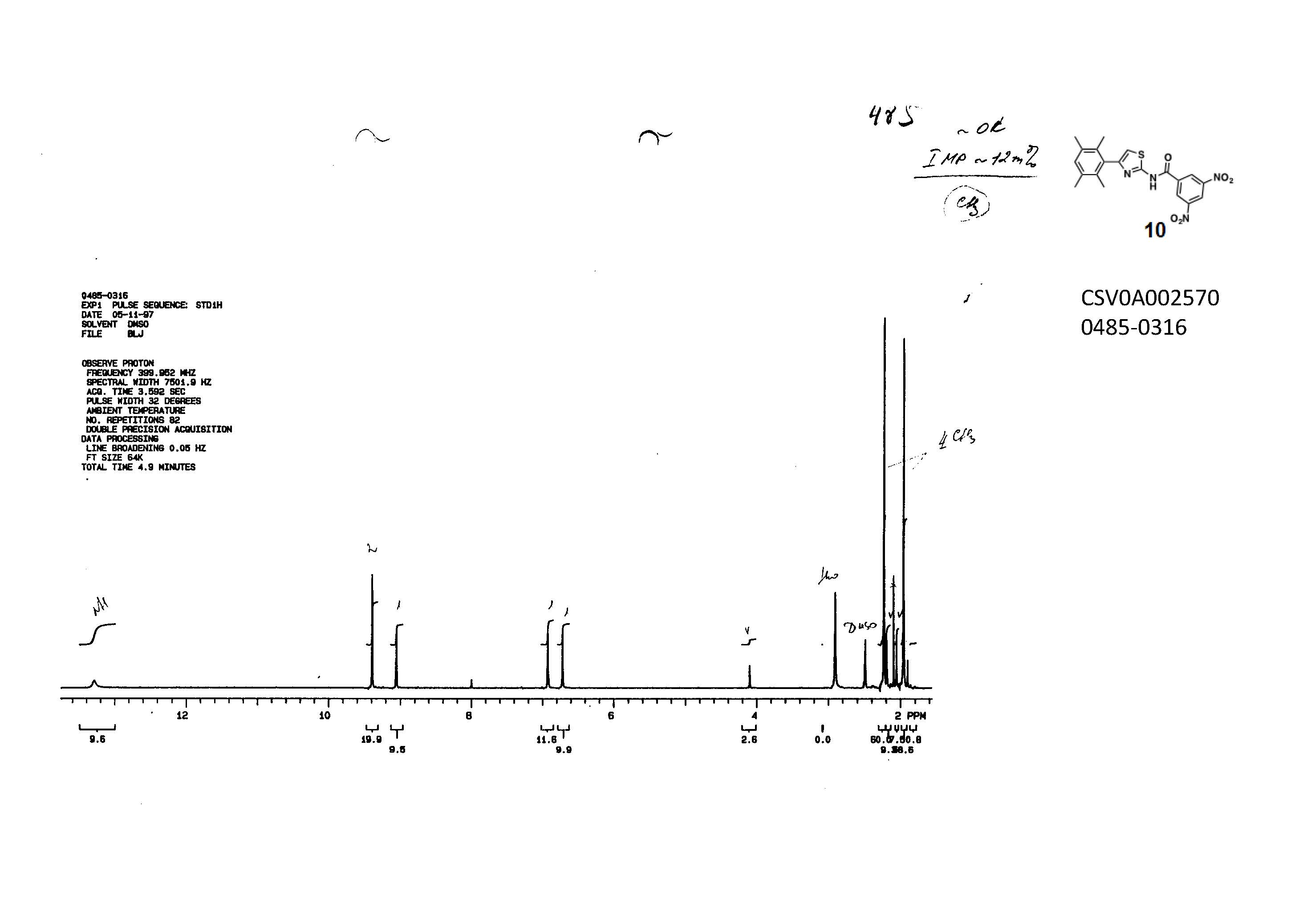


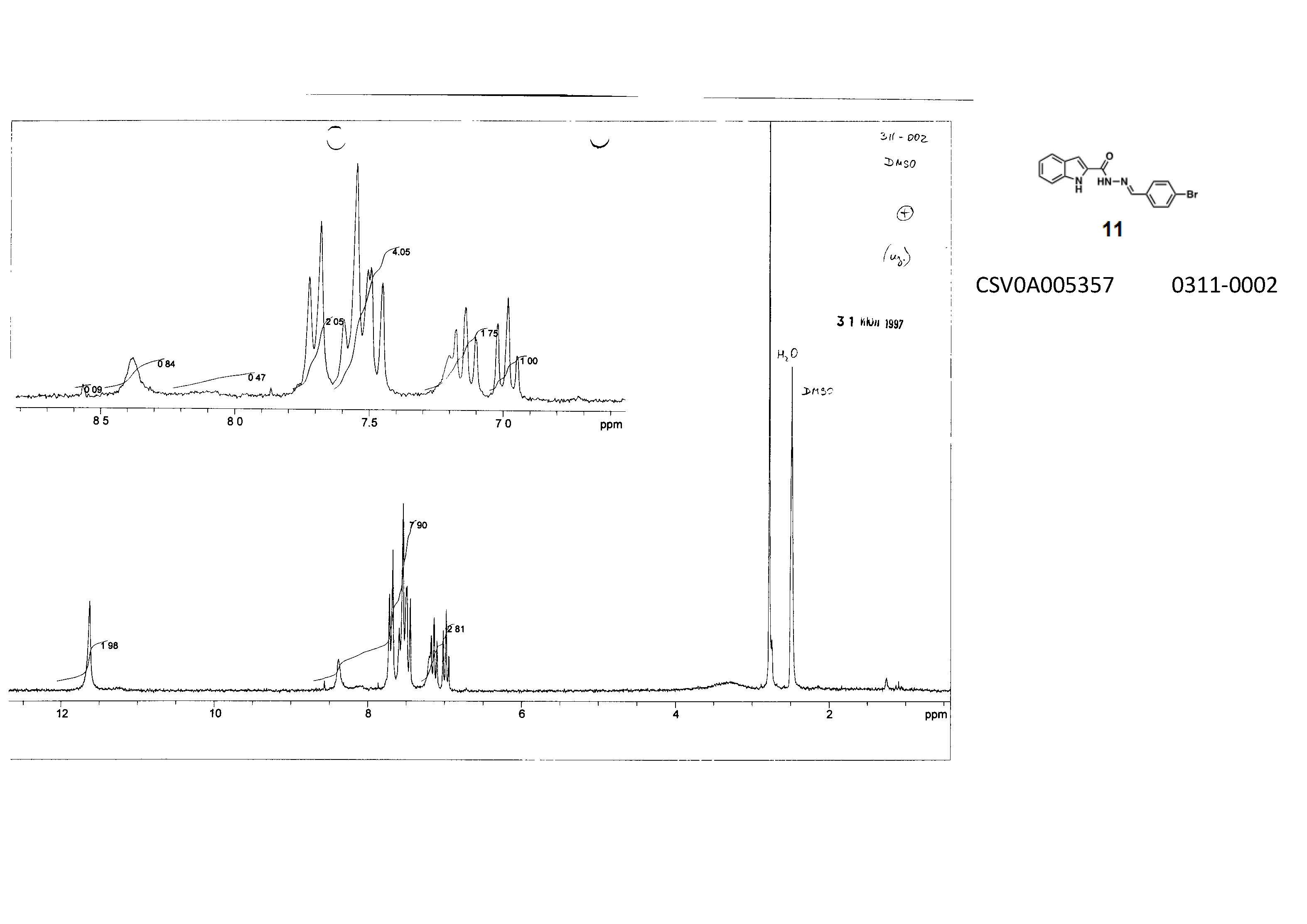


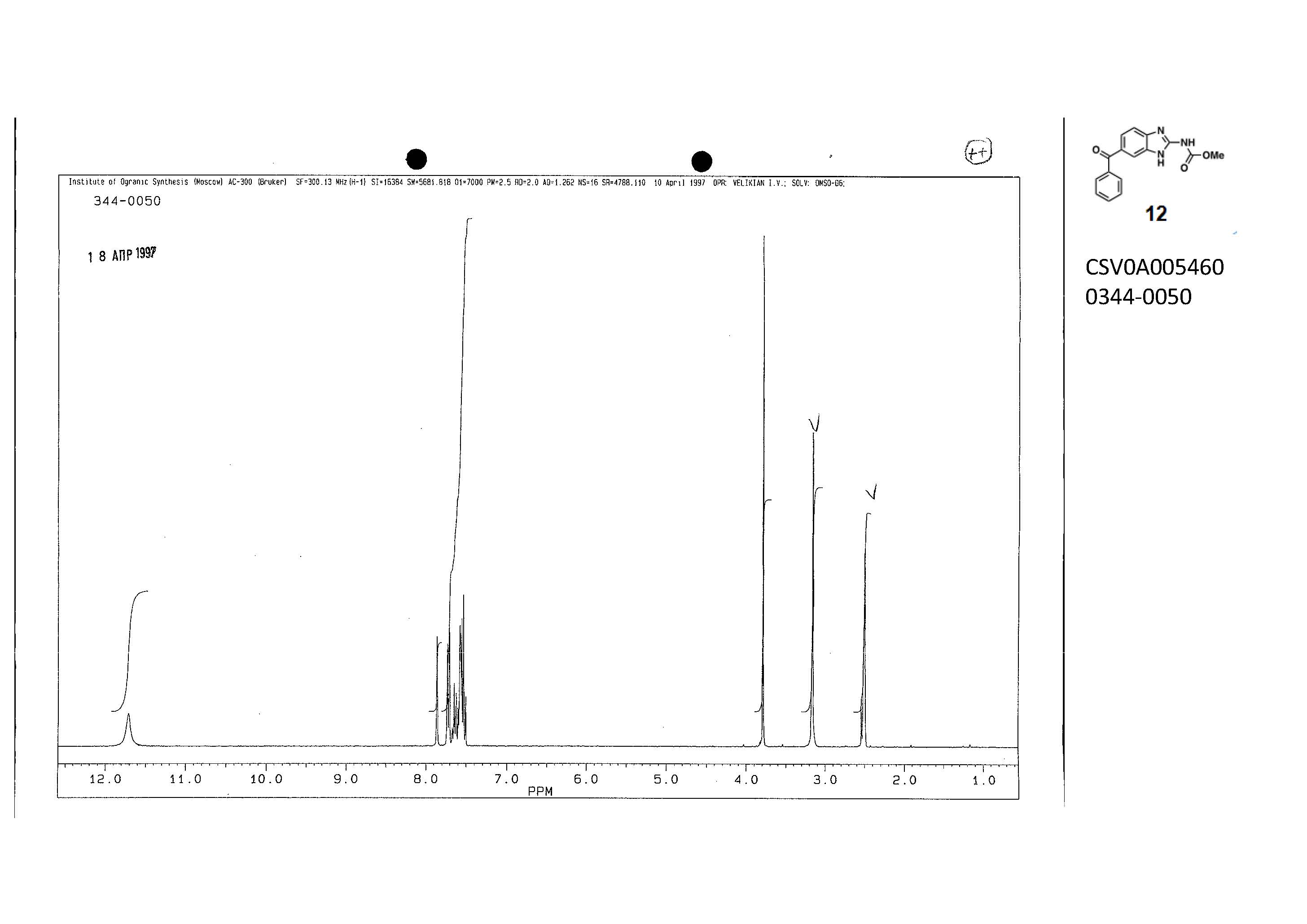


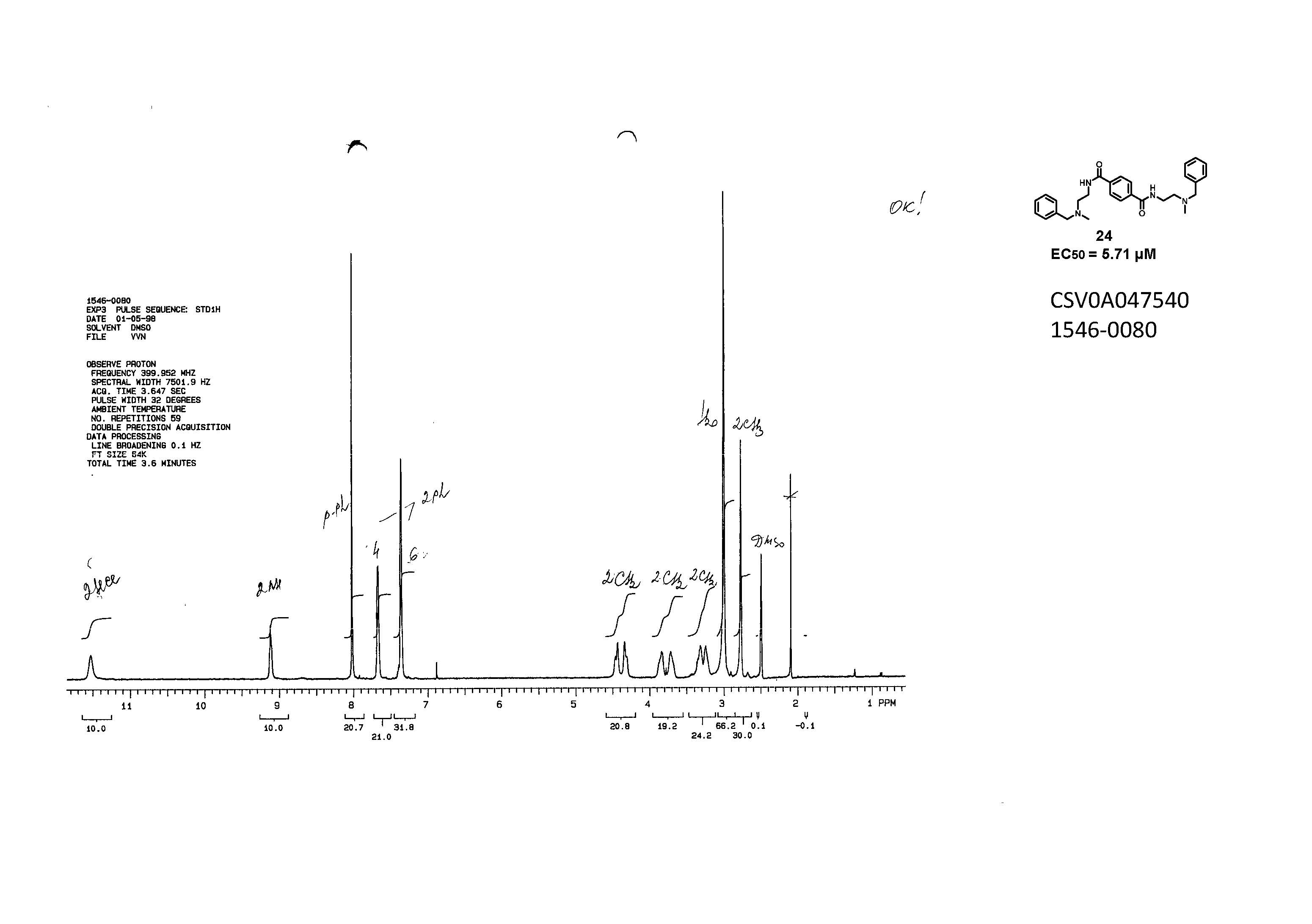


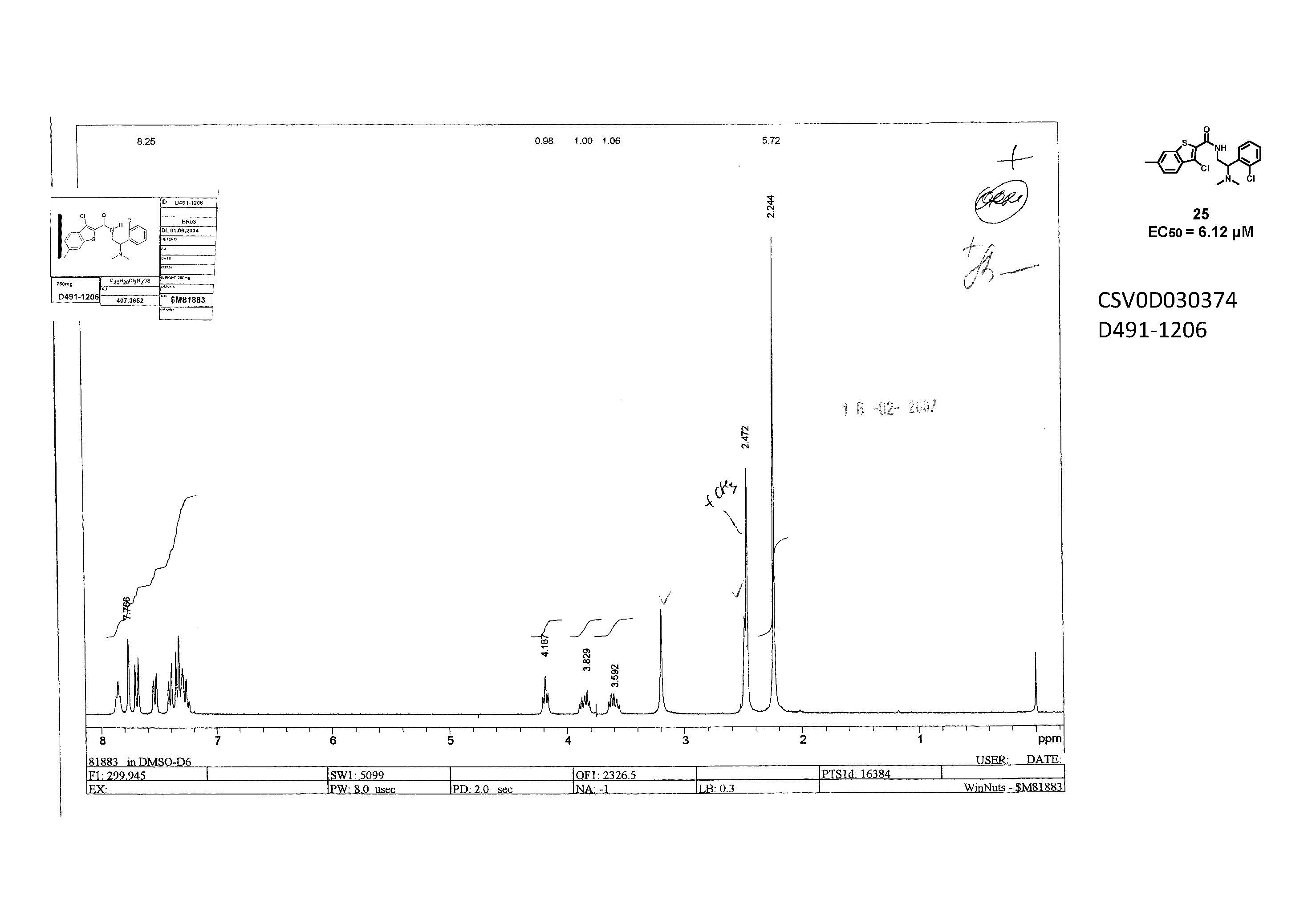


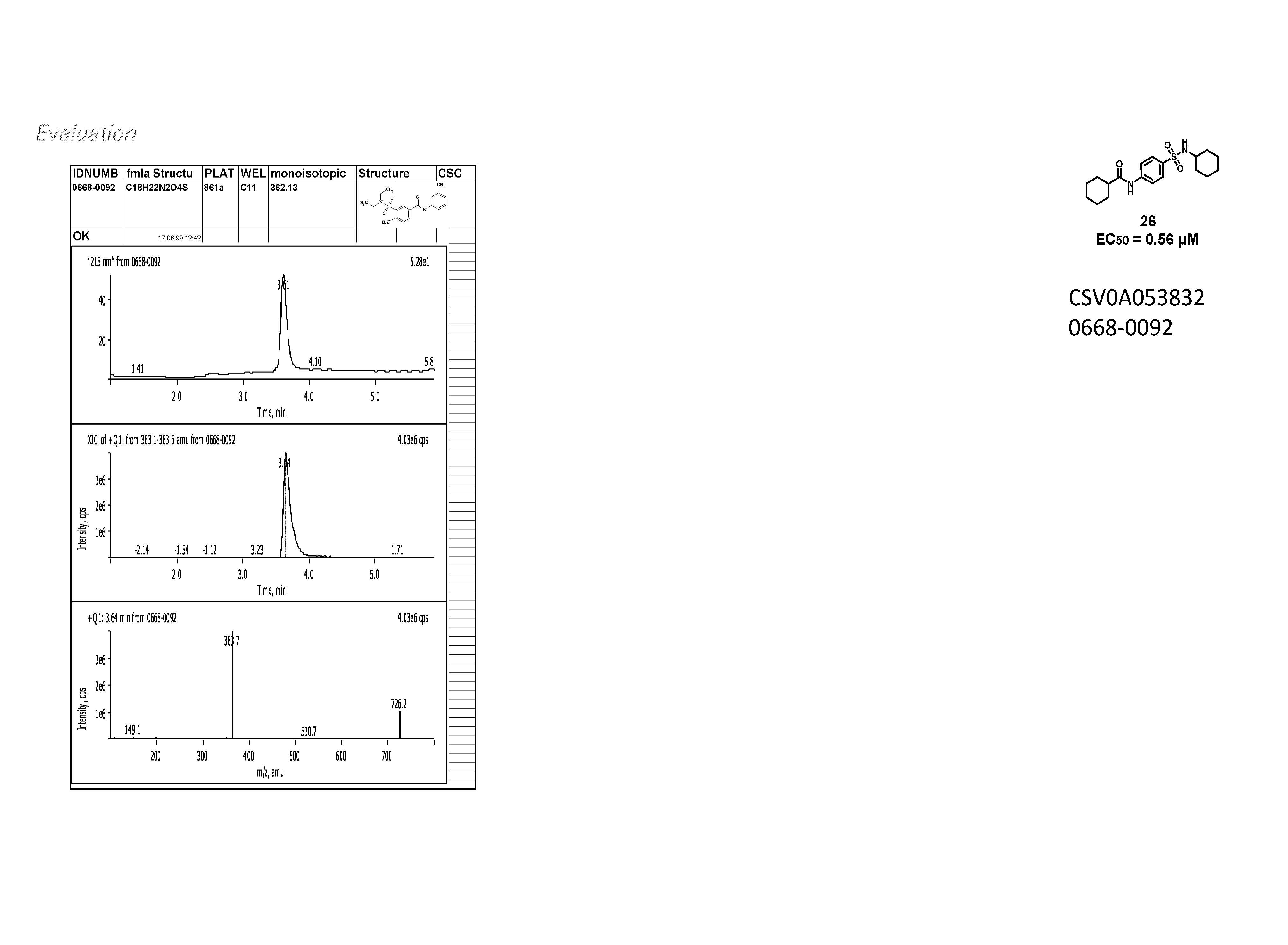


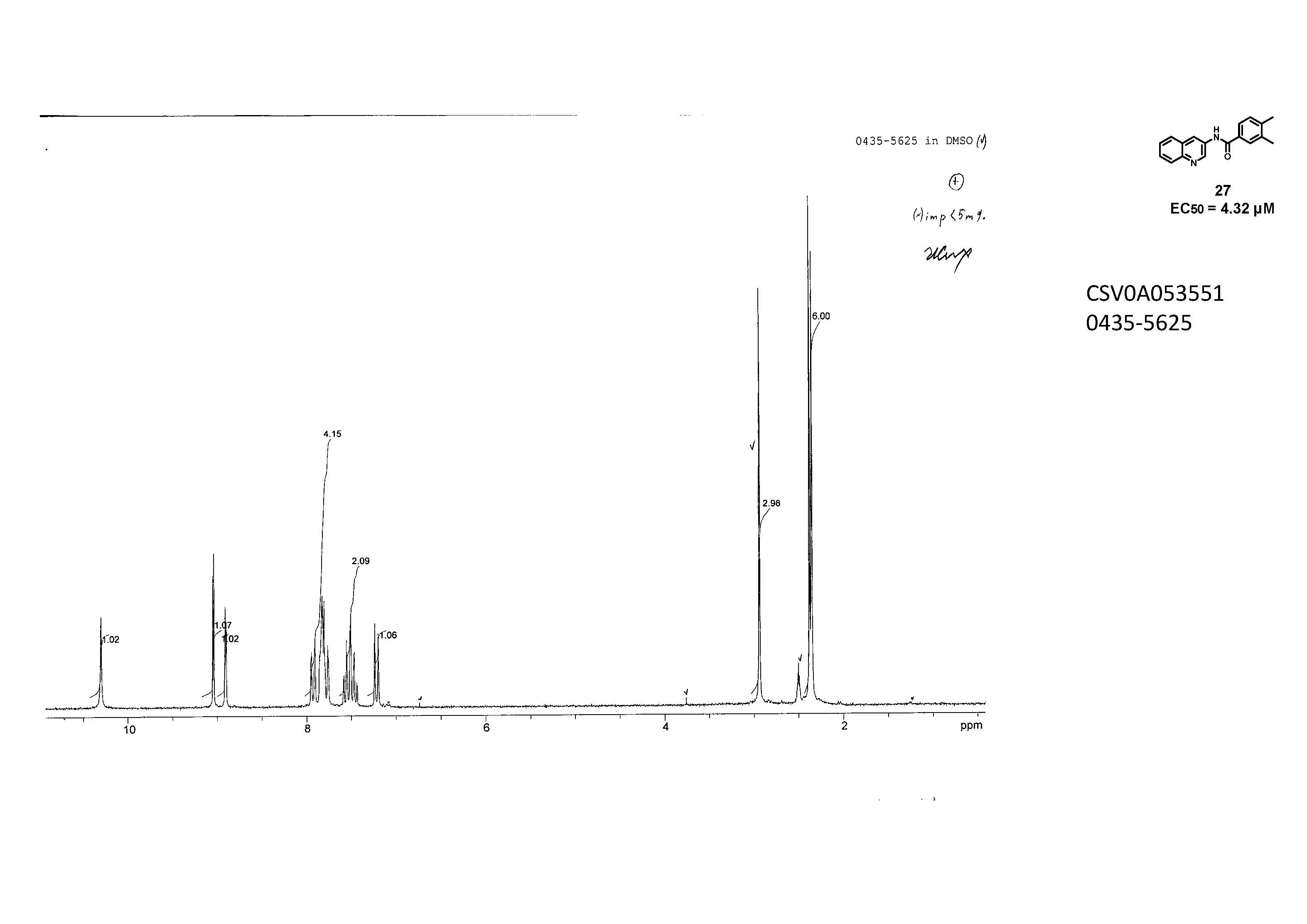


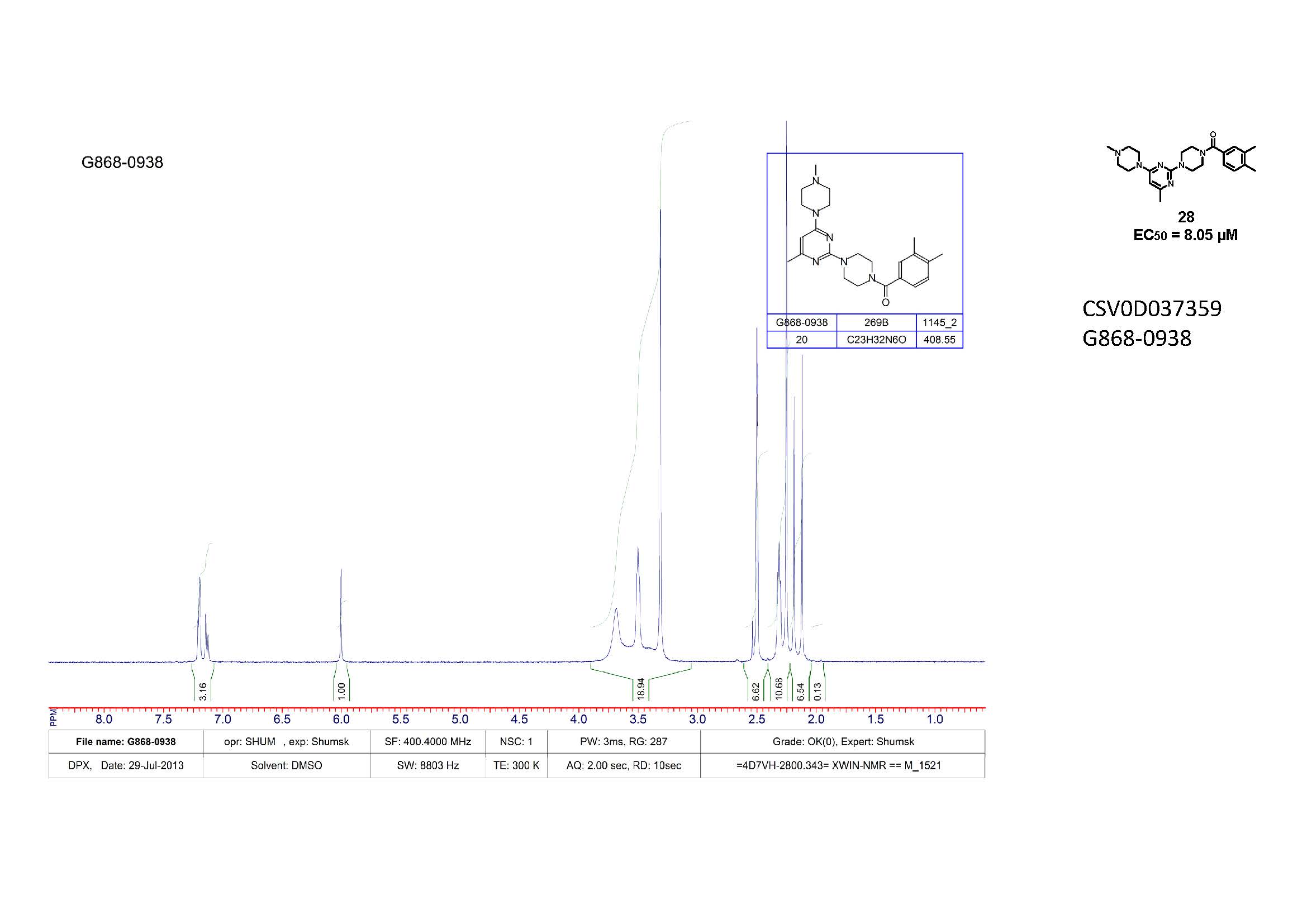


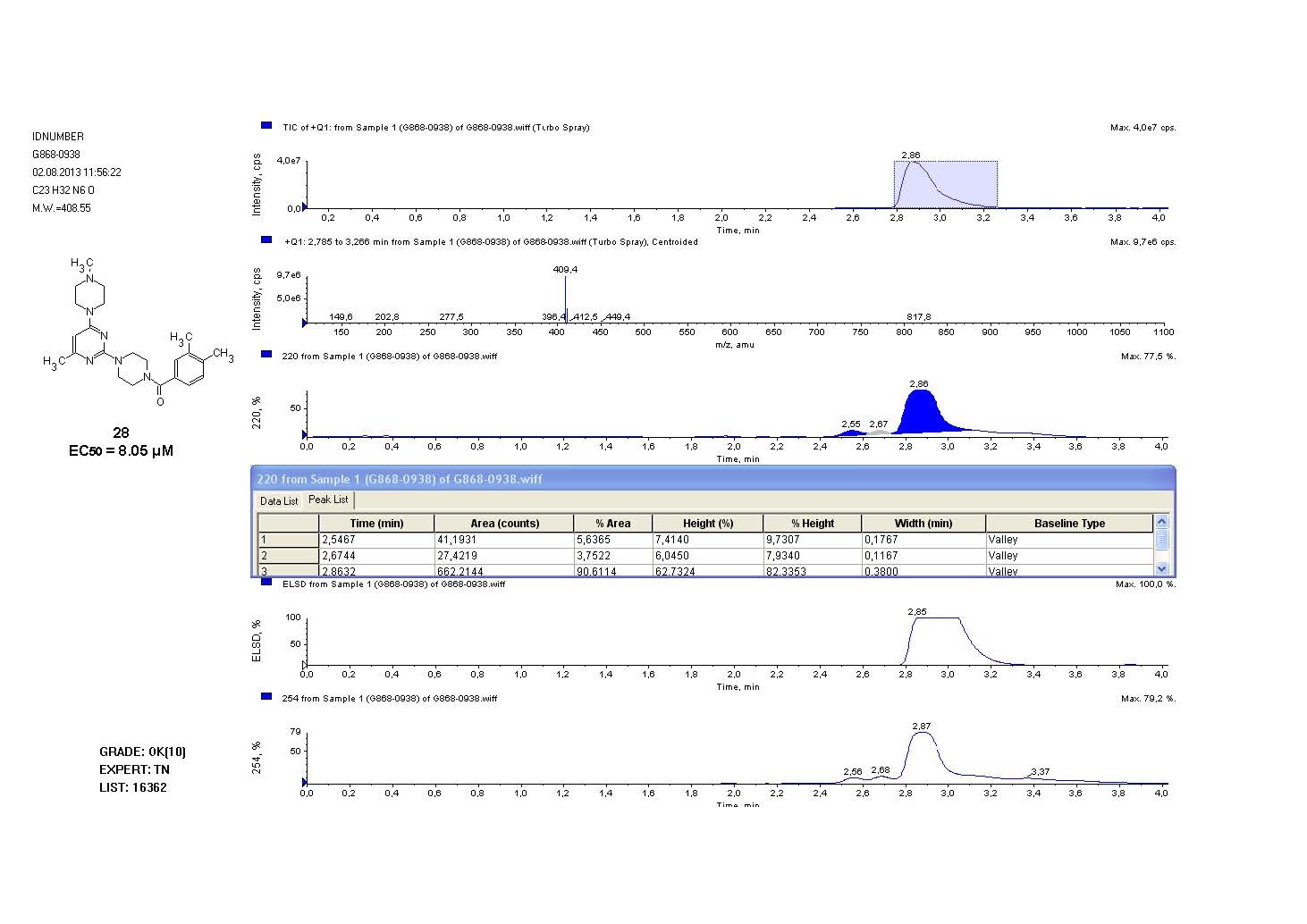

Supplement: Supplementary file 1 — Supplementary Information 1. [file 41598_2020_73681_MOESM1_ESM.docx]
